# Supplementary material for: An E2-E3 pair contributes to seed size control in grain crops
Source: Nat Commun. 2023 May 29;14:3091. doi: 10.1038/s41467-023-38812-y (PMC10226984; doi:10.1038/s41467-023-38812-y)
Supplement: Supplementary file 1 — Supplementary Information [file 41467_2023_38812_MOESM1_ESM.pdf]

Supplementary Information

An E2-E3 pair contributes to seed size control in grain crops

Authors: Sha Tang, Zhiying Zhao, Xiaotong Liu, Yi Sui, Dandan Zhang, Hui Zhi, Yuanzhu Gao, Hui Zhang, Linlin Zhang, Yannan Wang, Meicheng Zhao, Dongdong Li, Ke Wang, Qiang He, Renliang Zhang, Wei Zhang, Guanqing Jia, Wenqiang Tang, Xingguo Ye, Chuanyin Wu, Xianmin Diao

Correspondence: Xianmin Diao (Email: diaoxianmin@caas.cn)

This PDF file includes:

Supplementary Figures 1-16.

Supplementary Tables 1-6.

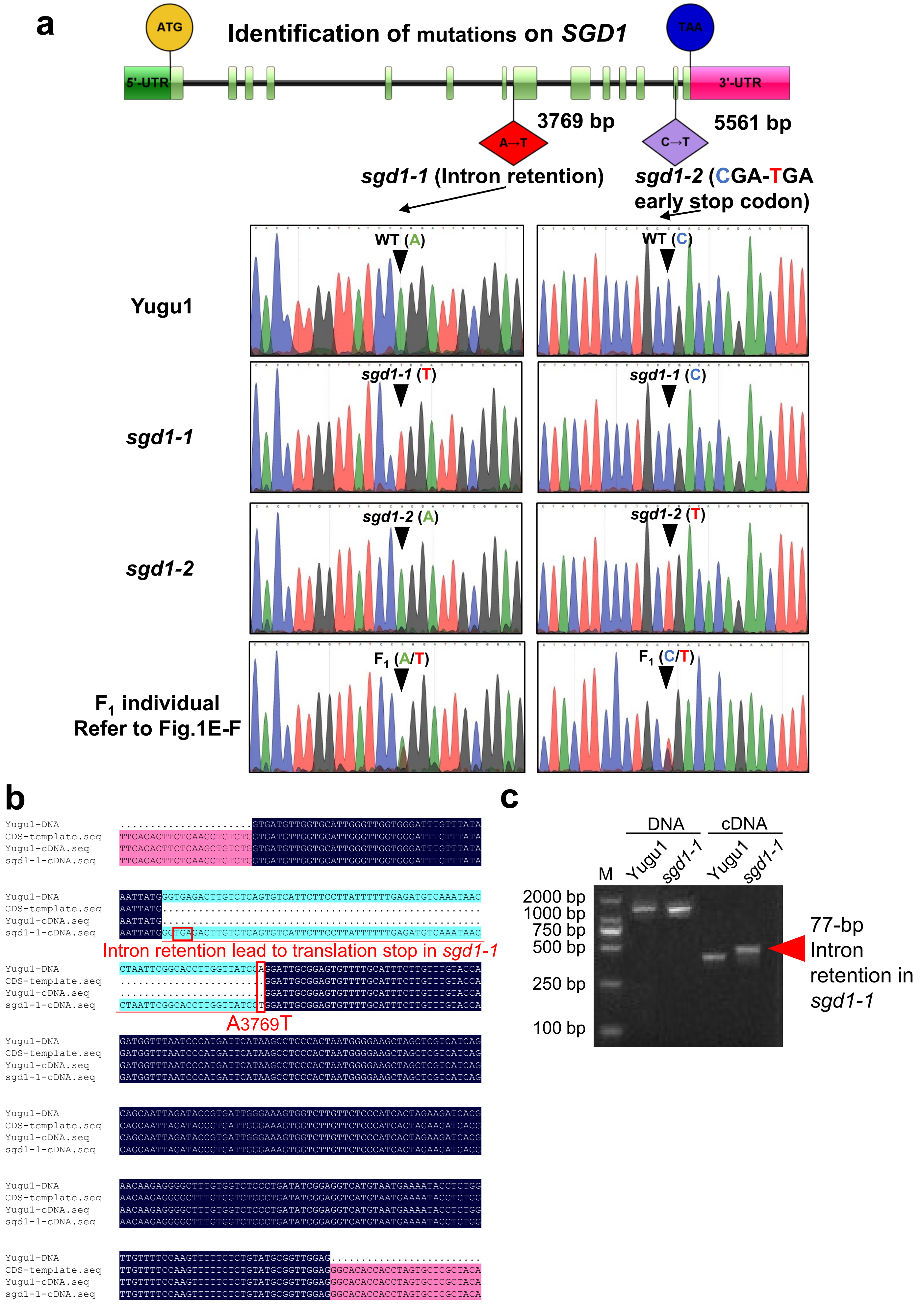

Supplementary Fig. 1 | Identification of *SGD1* mutations in different plant materials. a Identification of SNPs in Yugu1, mutants, and F_1 (*sgd1-1*×*sgd1-2*) individuals by Sanger sequencing. **b, c** Sequences alignments (**b**) and agarose gel electrophoresis (**c**) revealed a 77-bp intron retention in the *sgd1-1* mutant, leading to translation termination. This experiment was repeated three times independently with similar results.

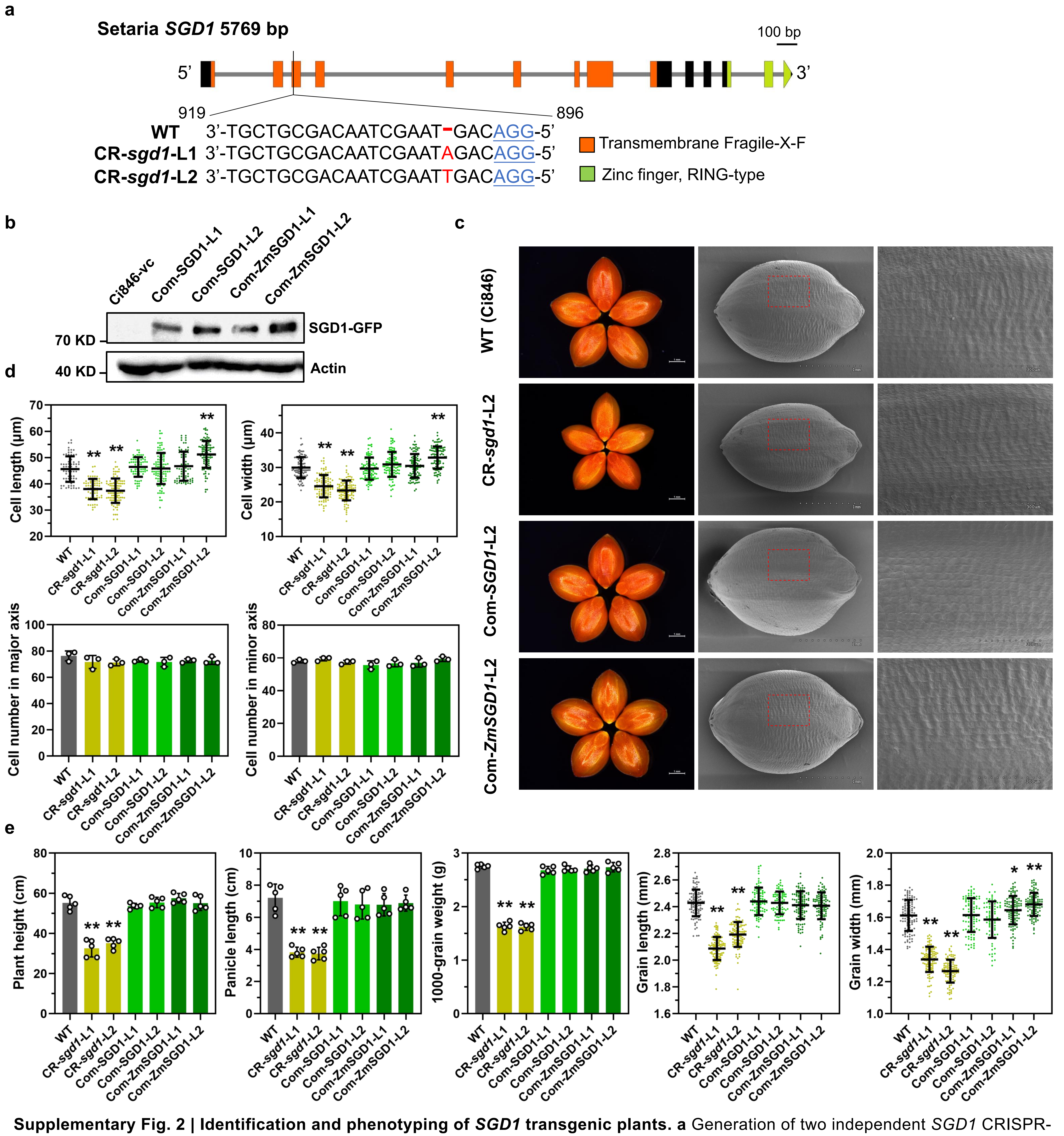

Supplementary Fig. 2 | Identification and phenotyping of *SGD1* transgenic plants. **a** Generation of two independent *SGD1* CRISPR-edited plant lines (*CR-sgd1*-L1, *CR-sgd1*-L2). The *SGD1* gene structure, position, and sequence of sgRNA are illustrated, the PAM motif is underlined, and edited sequences are highlighted in red. **b** Immunoblot analysis of SGD1-GFP and ZmSGD1-GFP in genetic complementation transgenic plants (Fig. 2) using an anti-GFP antibody. A wild-type (WT) line was used as a control. Actin was used as a loading control. This experiment was repeated three times independently with similar results. **c** Morphological features and scanning electron microscopy analysis of mature seeds of WT, *CR-sgd1*-L2, Com-*SGD1*-L2, and Com-*ZmSGD1*-L2 plants. Bar = 1 mm (first and second columns), Bar = 300 μm (third column). This experiment was repeated three times independently with similar results. **d** Cell length (n > 50), cell width (n > 50), and cell number in the major and minor axes (n = 5) in WT, *CR-sgd1*-L1, *CR-sgd1*-L2, Com-*SGD1*-L1, Com-*SGD1*-L2, Com-*ZmSGD1*-L1, and Com-*ZmSGD1*-L2 lines. **e** Plant height (n = 5), panicle length (n = 50), 1000-grain weight (n = 5), grain length (n > 100), and grain width (n > 100) in WT, *CR-sgd1*-L1, *CR-sgd1*-L2, Com-*SGD1*-L1, Com-*SGD1*-L2, Com-*ZmSGD1*-L1, and Com-*ZmSGD1*-L2. Data are means ± SD of n biological replicates. Significant differences were determined using unpaired two-sided Student's *t*-tests. $^{*}P < 0.05$, $^{**}P < 0.001$ vs. WT plants. n.s.: not statistically significant. Source data are provided as a Source Data file.

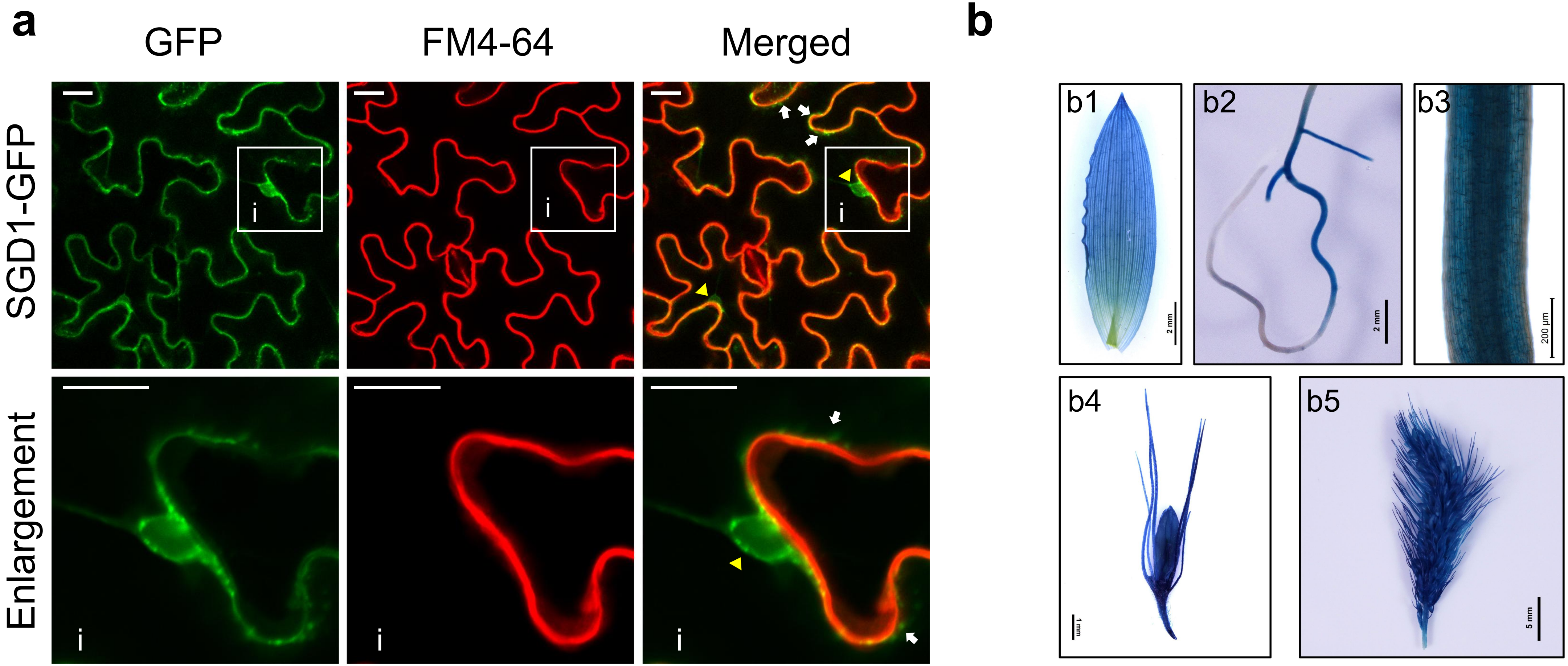

Supplementary Fig. 3 | Expression pattern and subcellular location of SGD1. a SGD1-GFP colocalized with the membrane marker FM4-64. The white arrows indicate the punctate signals of SGD1-GFP. The yellow arrows indicate endoplasmic reticulum signals. White boxes marked with i represent the magnification positions with enlarged views in the second row. Bar = 20 μm. **b** Histochemical staining of young leaf (**b1**), young root (**b2**), root elongation zone (**b3**), floret (**b4**), and milking panicle (**b5**) in *pSGD1::GUS* transgenic plants. These experiments in **a** and **b** were repeated three times independently with similar results.

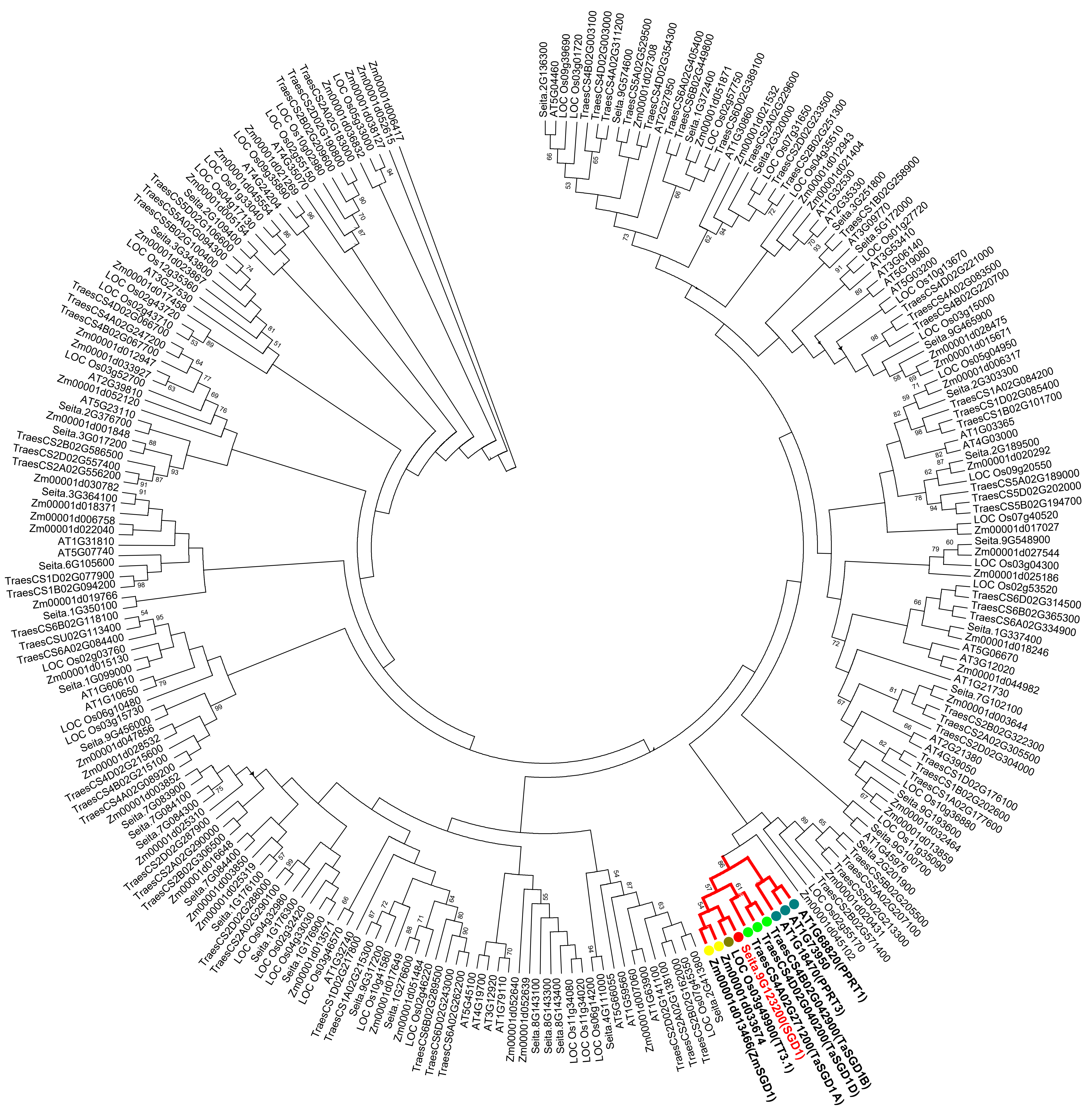

Supplementary Fig. 4 | Phylogenetic analysis of SGD1 and other E3s in *Arabidopsis thaliana*, *Setaria italica*, *Oryza sativa*, *Zea mays*, and *Triticum aestivum*. Full-length amino acid sequences of C3HC4 E3s (38 in *A. thaliana* [AT], 39 in *S. italica* [Seita], 40 in *O. sativa* [LOC_Os], 53 in *Z. mays* [Zm], and 70 in *T. aestivum* [Traes]). Sequences were aligned using MUSCLE in MEGA X software. Aligned sequences were used to construct an unrooted phylogenetic tree based on a maximum likelihood method after bootstrap analysis for 1000 replicates. The red branch represents SGD1 and orthologous proteins.

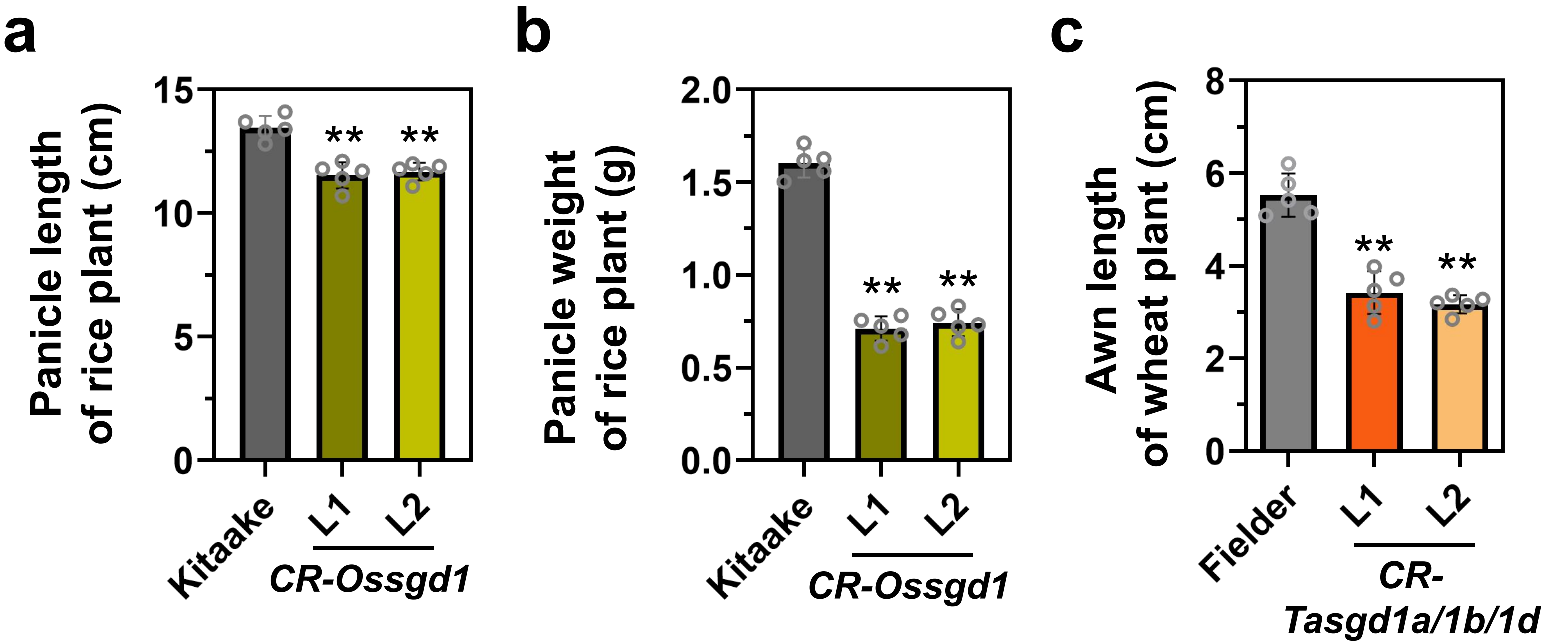

Supplementary Fig. 5 | Panicle traits in transgenic rice and wheat. a Panicle length (n = 5), **b** panicle weight (n = 5) in wild-type (WT) (KitaaKe), *CR-Ossgd1*-L1, and *CR-Ossgd1*-L2 rice plants. **c** Awn length (n = 5) in WT (Fielder), *CR-Tasgd1a/1b/1d*-L1, and *CR-Tasgd1a/1b/1d*-L2 wheat plants. Significant differences were determined using unpaired two-sided Student's *t*-tests. **P < 0.001 vs. WT plants. Error bars indicate mean ± SD. Source data are provided as a Source Data file.

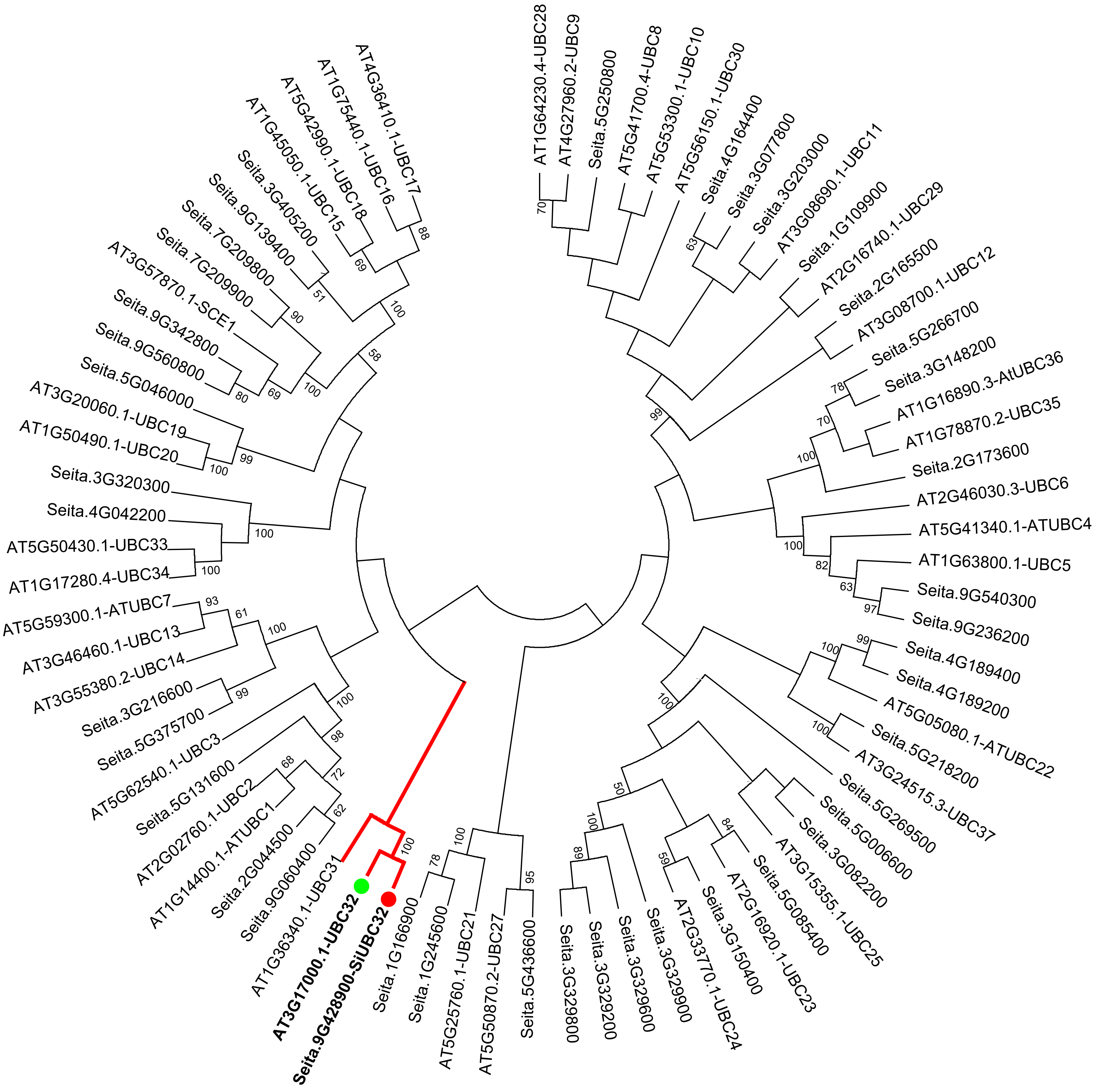

Supplementary Fig. 6 | Phylogenetic analysis of Arabidopsis and foxtail millet E2 enzymes. The complete amino acid sequences of 78 E2 enzymes were aligned using MUSCLE and analyzed phylogenetically using MEGA X software. An unrooted phylogenetic tree was constructed using the neighbor-joining method by bootstrap analysis with 1000 replicates. The clade containing UBC32 is highlighted in red.

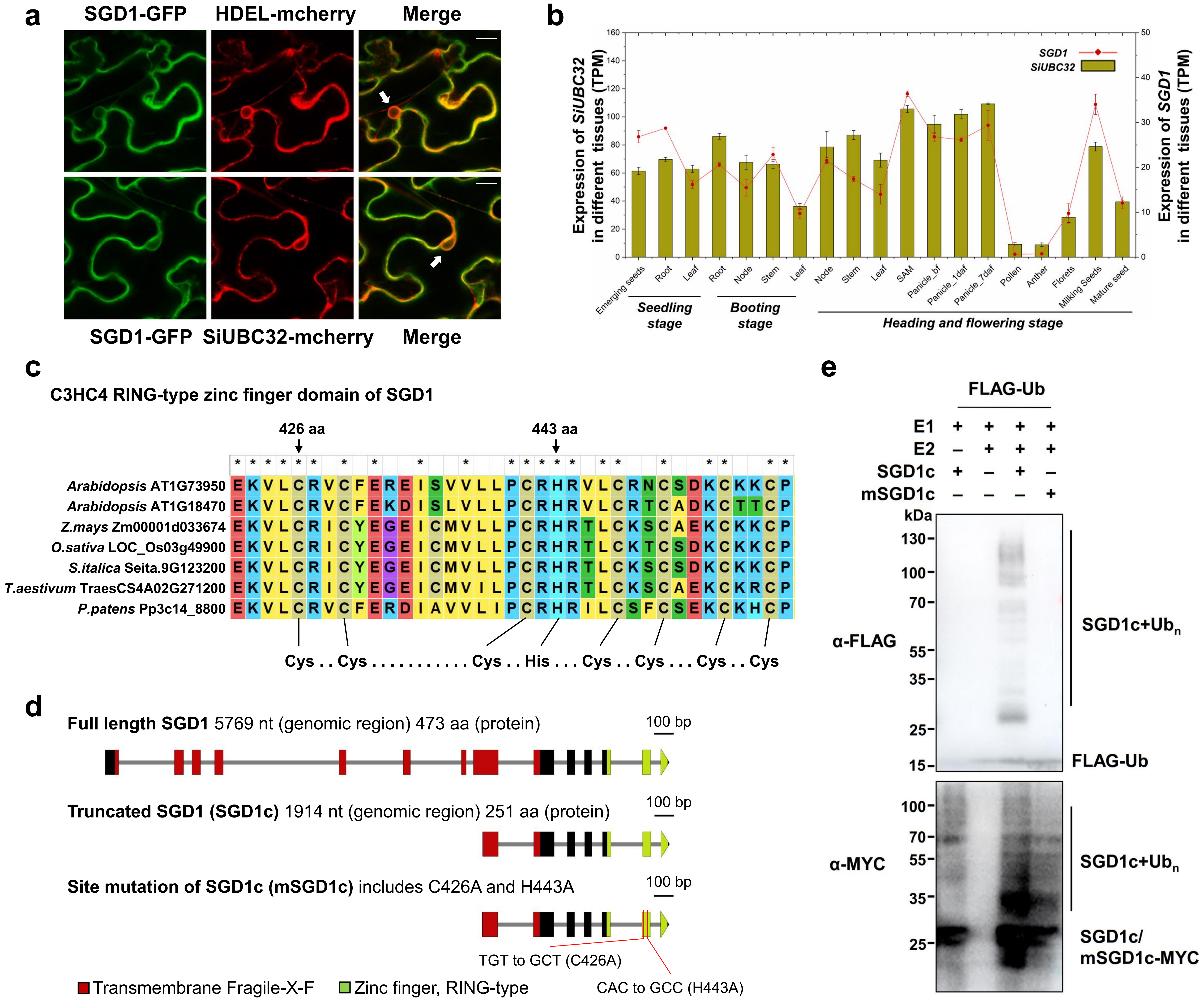

Supplementary Fig. 7 | Expression pattern and subcellular location of SiUBC32 and mSGD1 ligase activity assays. a SGD1-GFP colocalized with the ER marker HDEL-mCherry and SiUBC32-mCherry in *Nicotiana benthamiana* leaf cells. The white arrows indicate typical ER signals. Bar = 20 μm. This experiment was repeated three times independently with similar results. **b** Expression of *SiUBC32* and *SGD1* in foxtail millet tissues at the seedling, booting, heading, and flowering stages. n = 3 biological replications. Error bars indicate mean ± SD. The gene expression profile in 19 tissues of Yugu1 plants is shown in Supplementary Table S4. **c** Alignment of the C3HC4 RING-type zinc finger domain of the SGD1 protein family. The black arrows indicated conserved functional sites in the SGD1 RING finger domain. **d** Genomic structure of full-length SGD1, truncated SGD1 (SGD1c), and SGD1c with two mutations (C426A and H443A) in the RING domain (mSGD1c). Rectangles and lines represent exons and introns, respectively. **e** mSGD1 ligase activity assays. Lysates of *E. coli* strains expressing E1, E2, SGD1c-Myc, mSGD1c-Myc, and His-FLAG-Ub, or strains lacking one or two of these components. The activitives of SGD1c and mSGD1c were detected by Western blotting with an anti-Myc antibody. Ub conjugates were detected by Western blotting using an anti-FLAG antibody. This experiment was repeated three times independently with similar results. Source data are provided as a Source Data file.

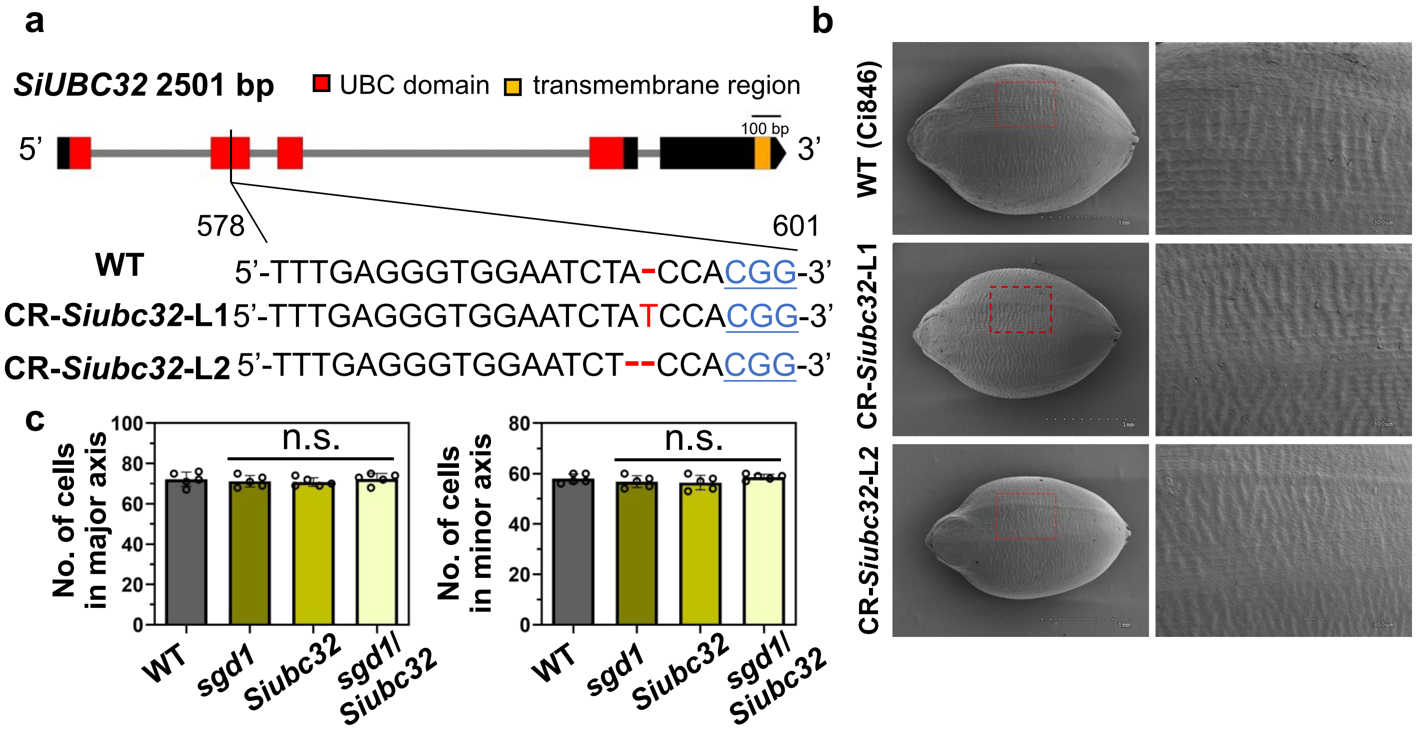

Supplementary Fig. 8 | Identification and phenotyping of CRISPR-edited *SiUBC32*. **a** Generation of two independent *SiUBC32* CRISPR-edited lines (*CR-Siubc32*-L1, *CR-Siubc32*-L2). The *SiUBC32* gene structure, position, and sgRNA sequence are illustrated, the PAM motif is underlined, and edited sequences are highlighted in red. **b** Scanning electron microscopy analysis of lemmas in wild-type (WT), *CR-Siubc32*-L1, and *CR-Siubc32*-L2 lines. Bar = 1 mm (left column), 300 μm (right column). **c** Cell count in the major and minor axes in WT, *CR-Siubc32*-L1, and *CR-Siubc32*-L2 lines. Data are means ± SD of five biological replicates. Significant differences were determined using unpaired two-sided Student's *t*-tests. n.s.: not statistically significant. Source data are provided as a Source Data file.

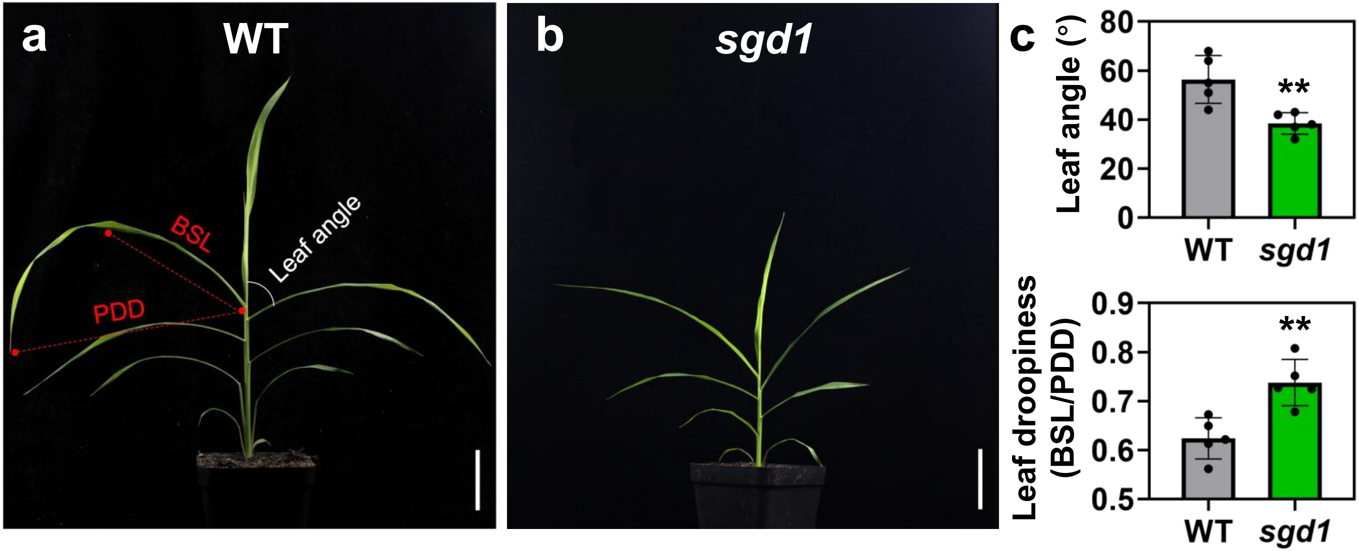

Supplementary Fig. 9 | Leaf angle and leaf drooping in *sgd1* mutants at the shooting stage. a Leaf architecture of wild-type (WT) plants grown in a growth chamber for 3 weeks under a 10-h light/14-h dark cycle. Bar = 5 cm. The white curve represents the leaf angle. The red dotted line represents BSL and PDD. Leaf drooping was calculated by the BSL to PDD ratio. **b** Leaf architecture of WT plants grown in a growth chamber for 3 weeks under a 10-h light/14-h dark cycle. Bar = 5 cm. **c** Leaf angle and leaf drooping in WT and *sgd1* plants (n = 5). Data were compared using unpaired two-sided Student's *t*-tests. Data are means ± SD. $^{**}P < 0.001$. Source data are provided as a Source Data file.

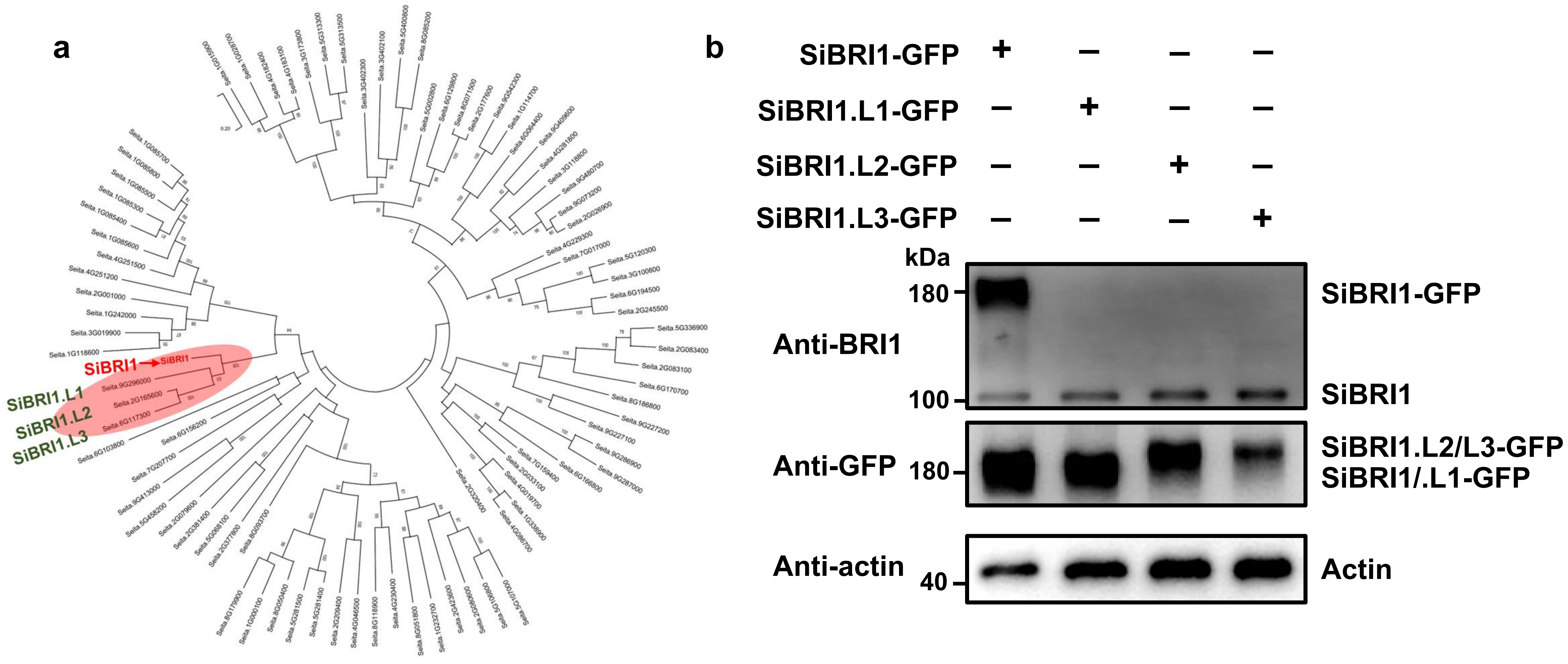

Supplementary Fig. 10 | Phylogenetic analysis of BRI1-related LRR proteins and the specificity of anti-SiBRI1 antibodies. a Phylogenetic analysis of Setaria italica BRI1 and its homologs. The red font represents SiBRI1, and the green font represents SiBRI1.L1, SiBRI1.L2, and SiBRI1.L3, which were highly similar to SiBRI1. **b** Specificity of anti-SiBRI1 antibodies. The coding sequences of SiBRI1, SiBRI.L1, SiBRI.L2, SiBRI.L3 were cloned into the pCAMBIA1305-eGFP vector and transfected into foxtail millet protoplasts. SiBRI1-GFP, SiBRI1.L1-GFP, SiBRI1.L2-GFP, and SiBRI1.L3-GFP were detected by anti-BRI1 and anti-GFP antibodies, respectively. This experiment was repeated three times independently with similar results. Actin was used as a loading control. Source data are provided as a Source Data file.

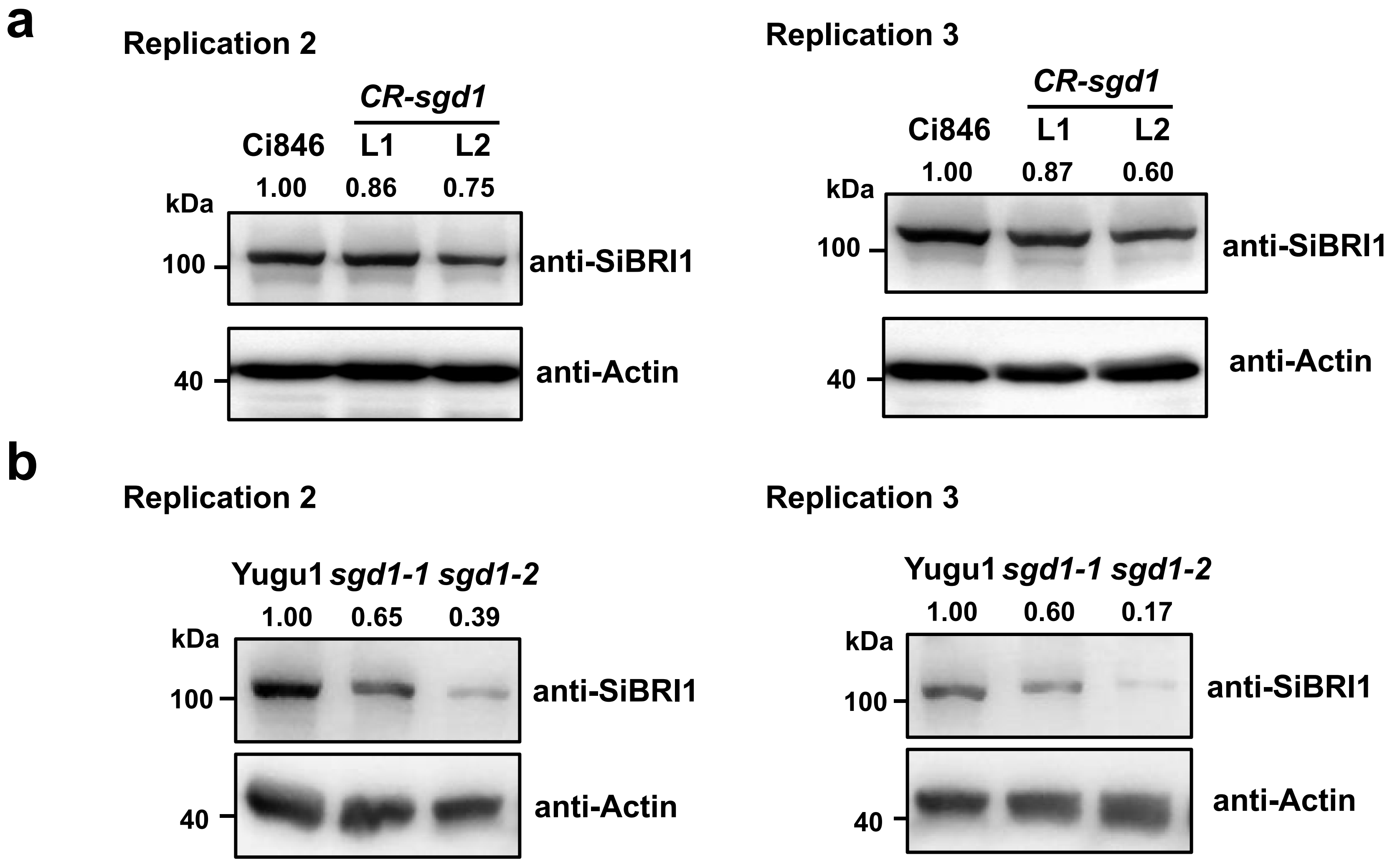

Supplementary Fig. 11 | Analysis of SiBRI1 stability in *sgd1* mutants. a Biological replicates 2 and 3 for SiBRI1 stability measurements in Ci846, *CR-sgd1*-L1, and *CR-sgd1*-L2 plants. Biological replicate 1 is shown in Fig. 6i. **b** Biological replicates 2 and 3 for SiBRI1 stability measurements in Yugu1, *sgd1-1*, and *sgd1-2* lines. Biological replicate 1 is shown in Fig. 6j. SiBRI1 in foxtail millet seedling leaves was detected by immunoblotting with an anti-SiBRI1 antibody. The ratio of SiBRI1 to actin is shown above the blots. Source data are provided as a Source Data file.

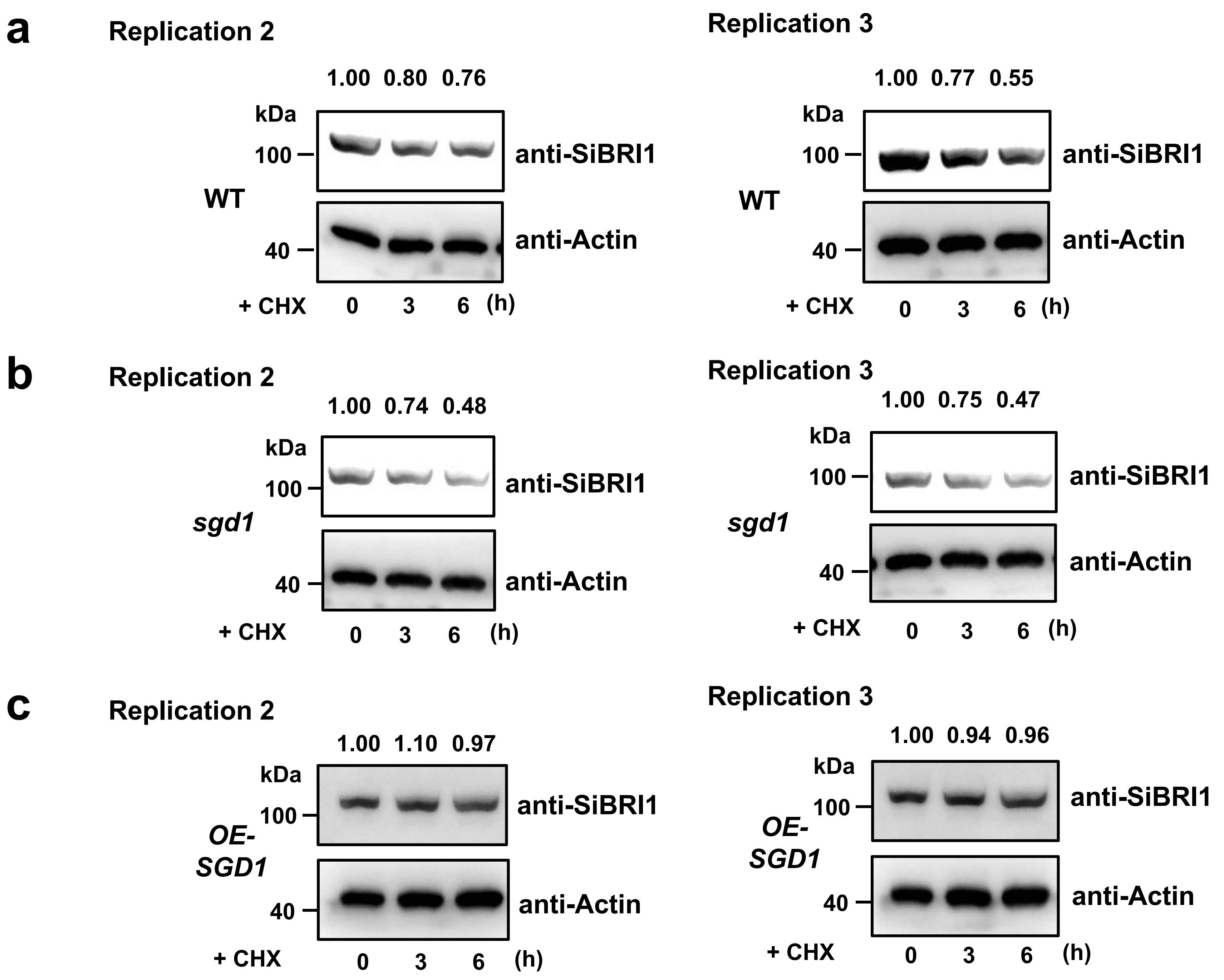

Supplementary Fig. 12 | SiBRI1 abundance after CHX treatment. a Biological replicates 2 and 3 for measurement of SiBRI1 abundance in wild-type plants in response to cycloheximide (CHX) treatment. Biological replicate 1 is shown in Fig. 6k. **b** Biological replicates 2 and 3 for measurement of SiBRI1 abundance in *sgd1* mutants (*CR-sgd1*-L1) in response to CHX treatment. Biological replicate 1 is shown in Fig. 6m. **c** Biological replicates 2 and 3 for measurement of SiBRI1 abundance in *OE-SGD1* plants in response to CHX treatment. Biological replicate 1 is shown in Fig. 6o. Fourteen-day-old Ci846, *sgd1*, and *OE-SGD1* seedling leaves were treated with 100 μM CHX for 0, 3, and 6 h. SiBRI1 was detected by immunoblotting with an anti-SiBRI1 antibody. Actin was used as a loading control. Source data are provided as a Source Data file.

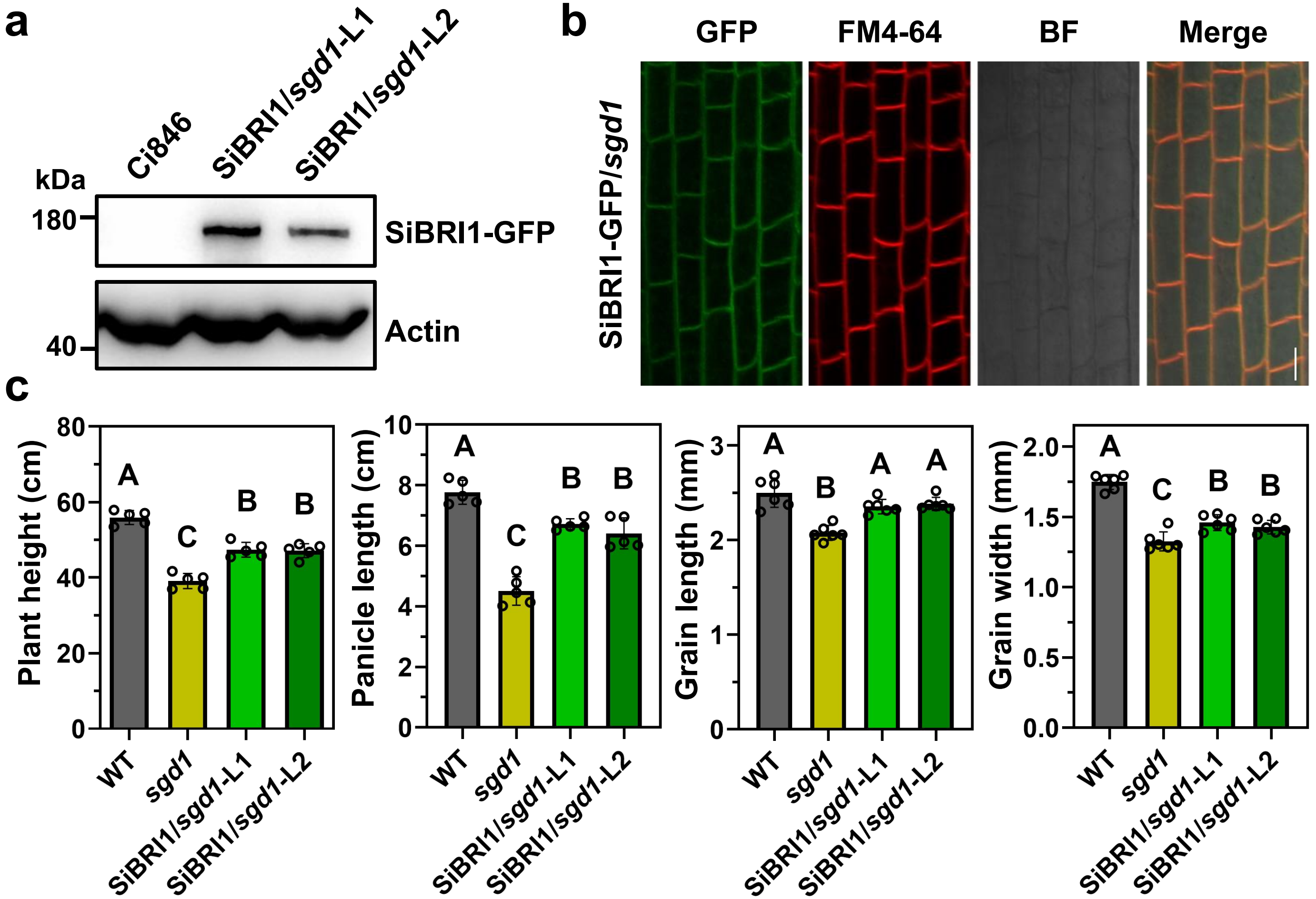

Supplementary Fig. 13 | Identification and phenotyping of SiBRI1/*sgd1* transgenic plants. a Immunoblot analysis of SiBRI1-GFP in genetic complementation transgenic plants (SiBRI1/*sgd1*-L1 and SiBRI1/*sgd1*-L2) (Fig. 6) using an anti-GFP antibody. A wild-type (WT) line was used as a control. Actin served as a loading control. **b** Confocal images showing the localization of SiBRI1-GFP. FM4-64 was used as a membrane marker. The roots of 3-day-old dark-grown SiBRI1/*sgd1* seedlings were used for observation. Bar = 20 μm. These experiments in **a** and **b** were repeated three times independently with similar results. **c** Plant height (n = 5), panicle length (n = 5), and grain length and width (n = 6) in WT, *sgd1*, SiBRI1/*sgd1*-L1, and SiBRI1/*sgd1*-L2 plants. Data are means ± SD of n biological replicates. Lowercase letters indicate significant differences ($P < 0.05$, one-way analysis of variance with Tukey's multiple comparisons test). Source data are provided as a Source Data file.

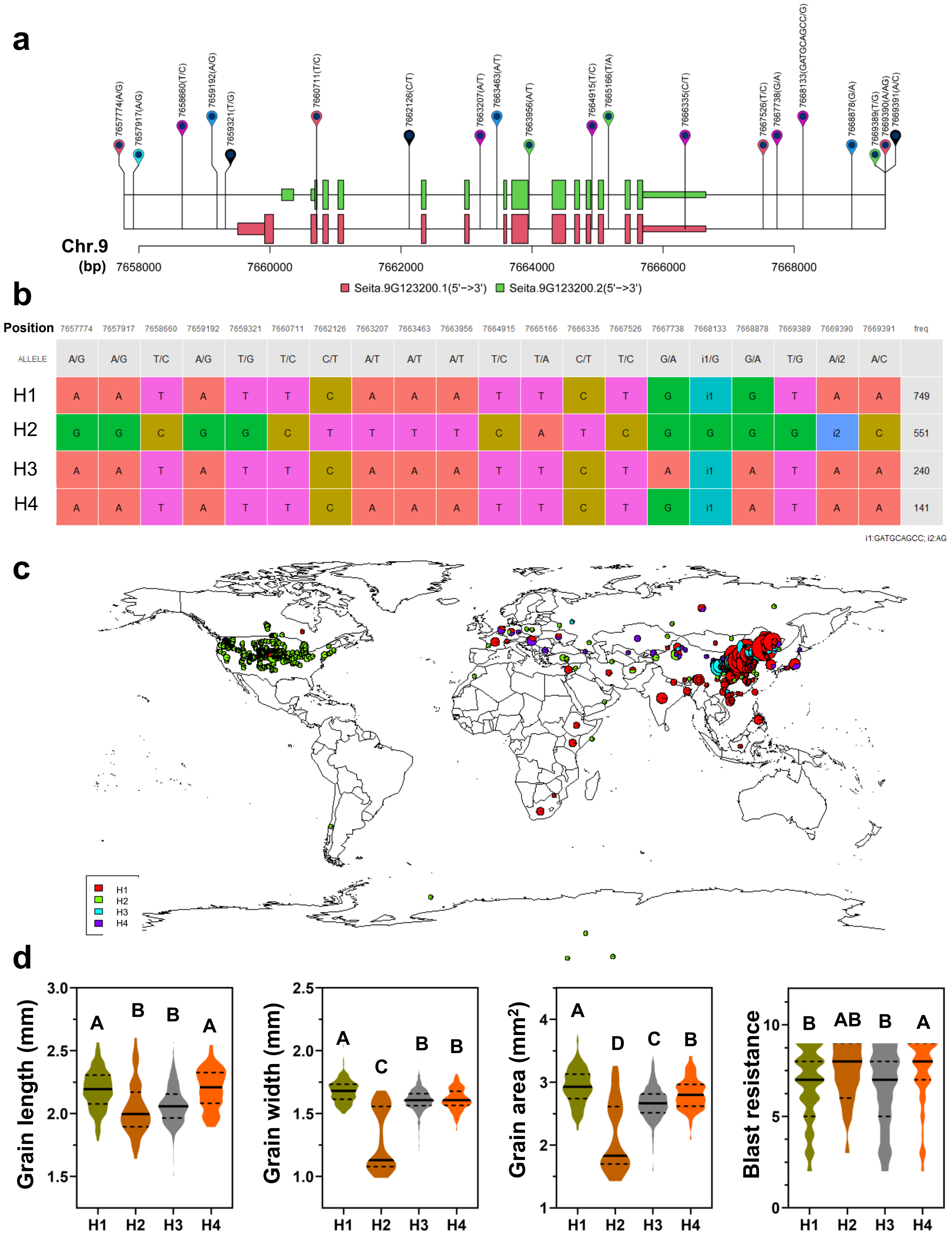

Supplementary Fig. 14 | Haplotype analysis of *SGD1*. a Variability in the *SGD1* gene. **b** Genomic variation in *SGD1* haplotypes. i: insertions or deletions (InDel); freq: number of foxtail millet varieties belonging to each haplotype. **c** Geographical distribution of *SGD1* haplotypes in 1681 *Setaria* germplasms. The circle size indicates the number of varieties. Each color represents a haplotype. The H1 haplotype was enriched in the varieties grown in the main production region. **d** Major agronomic traits (grain length, grain width, grain area, and blast resistance) in 960 of 1681 *Setaria* germplasms by *SGD1* haplotype. Uppercase letters indicate significant differences ($P < 0.05$, one-way analysis of variance with Tukey's multiple comparisons test). Source data are provided as a Source Data file.

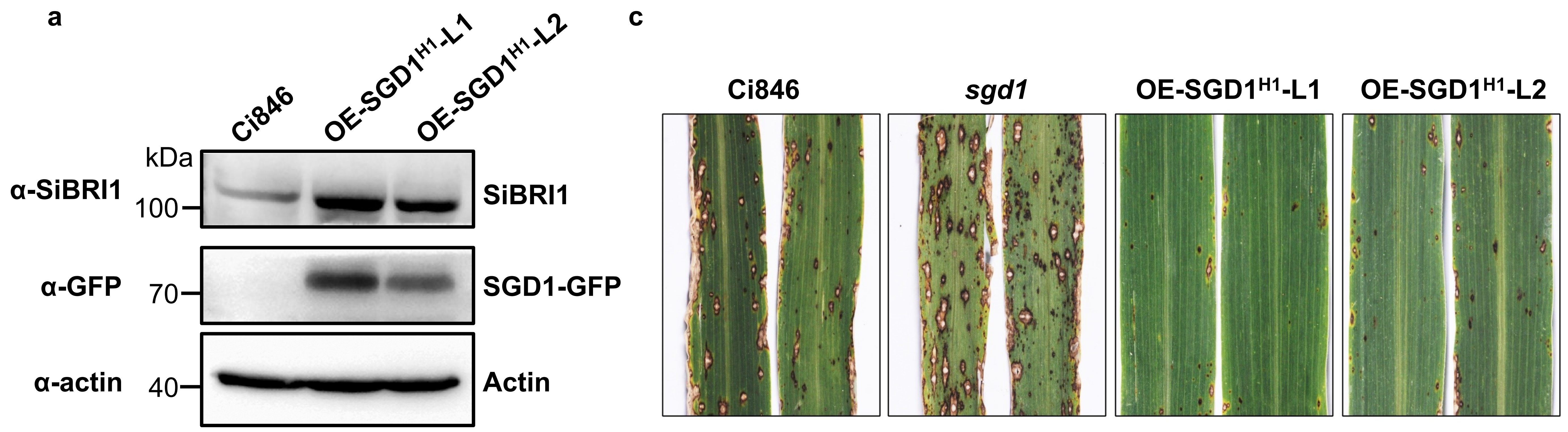

b

Agronomic trait	Ci846	*sgd1*	OE-*SGD1*^{H1}-L1	OE-*SGD1*^{H1}-L2	*n*
Panicle length (cm)	19.26±0.91	12.24±0.68**	21.10±1.14*	20.80±1.11*	5
Panicle width (cm)	2.13±0.04	1.54±0.03**	2.16±0.06	2.13±0.05	5
Panicle weight (g)	10.30±0.54	4.26±0.50**	11.62±0.85*	11.20±0.41*	5
1000-grain weight (g)	2.69±0.05	1.63±0.04**	3.07±0.06**	2.96±0.04**	5
Grain area (mm^2)	3.03±0.22	2.20±0.16**	3.40±0.19**	3.34±0.19**	170
Grain length (mm)	2.43±0.09	2.09±0.09**	2.54±0.09**	2.65±0.11**	170
Grain width (mm)	1.59±0.11	1.34±0.08**	1.71±0.08**	1.61±0.09	170

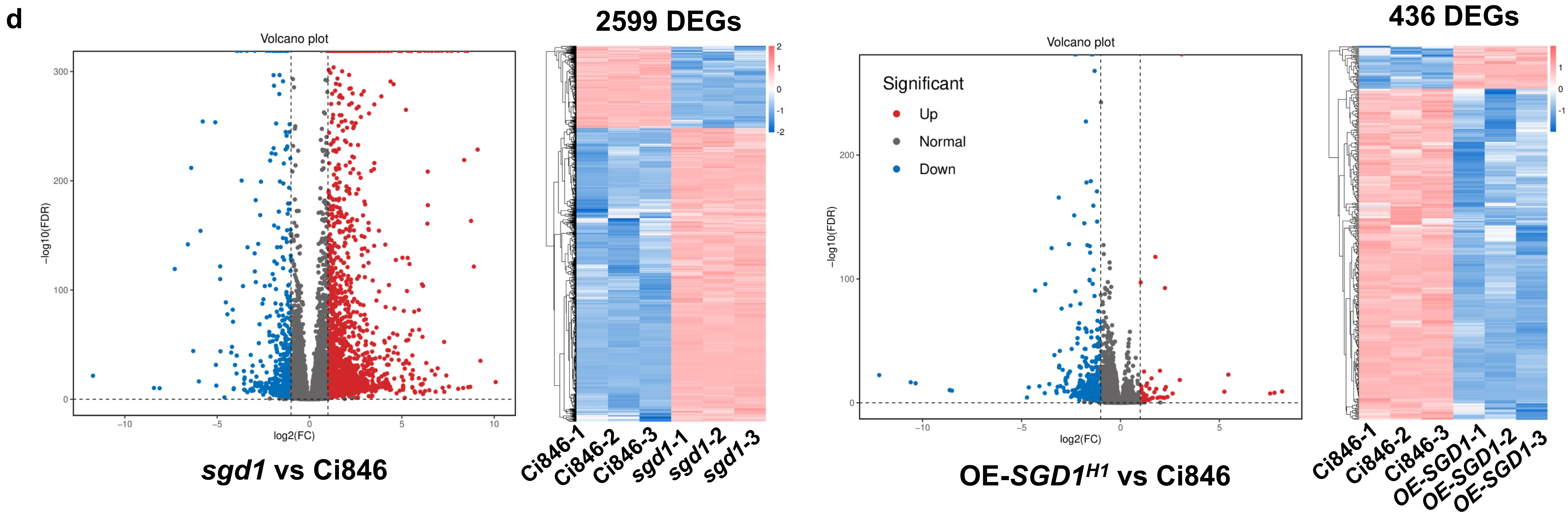

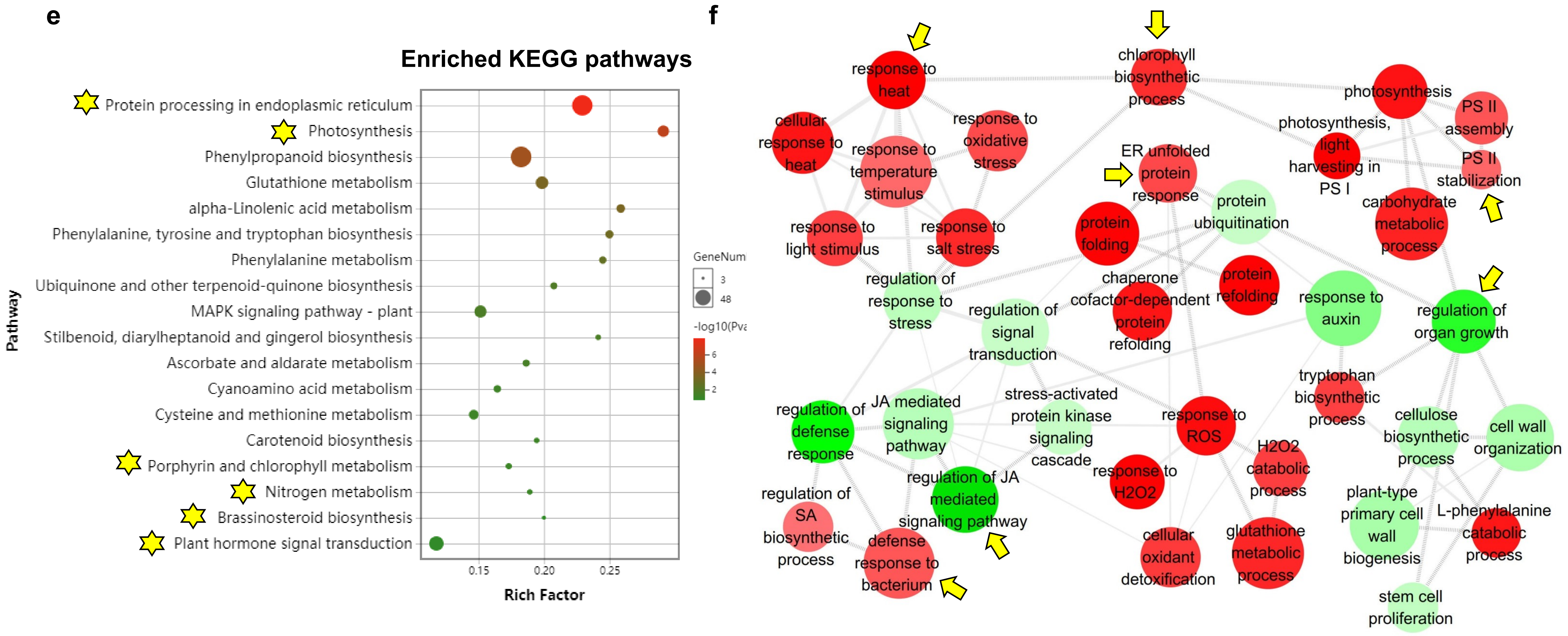

Supplementary Fig. 15 | Overexpression of the elite haplotype of *SGD1* increased grain yield and blast disease resistance. a Immunoblot analysis of SGD1-GFP and SiBRI1 in *SGD1*^{H1}-overexpressing plants (Fig. 7) using antibodies against GFP and SiBRI1. Actin was used as a loading control. Fourteen-day-old seedlings harvested from WT (Ci846) plants and plants overexpressing *SGD1*^{H1} (*OE-SGD1*^{H1}-L1 and *OE-SGD1*^{H1}-L2) were used for immunoblots. SGD1^{H1} overexpression enhanced SiBRI1 protein stability in vivo. This experiment was repeated three times independently with similar results. **b** Grain yield traits in Ci846, *sgd1*, *OE-SGD1*^{H1}-L1, and *OE-SGD1*^{H1}-L2 lines. Data are means ± SD of n biological replicates. Significant differences were determined using unpaired two-sided Student's *t*-tests. *P < 0.05, **P < 0.01 vs. Ci846 plants. **c** Disease resistance in *SGD1*^{H1}-overexpressing plants. Ci846, *sgd1*, *OE-SGD1*^{H1}-L1, and *OE-SGD1*^{H1}-L2 plants grown in the field were inoculated with the *Pyricularia setariae* race HN-1 at the eight-leaf stage. Blast disease phenotype was analyzed 2 weeks after inoculation. **d** Differentially expressed genes (DEGs) in *sgd1* and *OE-SGD1*^{H1} plants relative to Ci846. The number of DEGs is indicated by the volcano plot. Gene expression profiles are indicated by heatmap. **e** Enriched KEGG pathways in *sgd1* (vs. Ci846) and *OE-SGD1*^{H1} (vs. Ci846). Circle size indicates the number of DEGs belonging to each pathway. The colour gradient indicates enriched *P*-value. **f** Gene ontology analysis of biological processes enriched in DEGs in *sgd1* vs. Ci846 (red) and *OE-SGD1*^{H1} vs. Ci846 (green). Circle size indicates the number of DEGs belonging to each process. The colour gradient indicates enriched *P*-value. Source data are provided as a Source Data file.

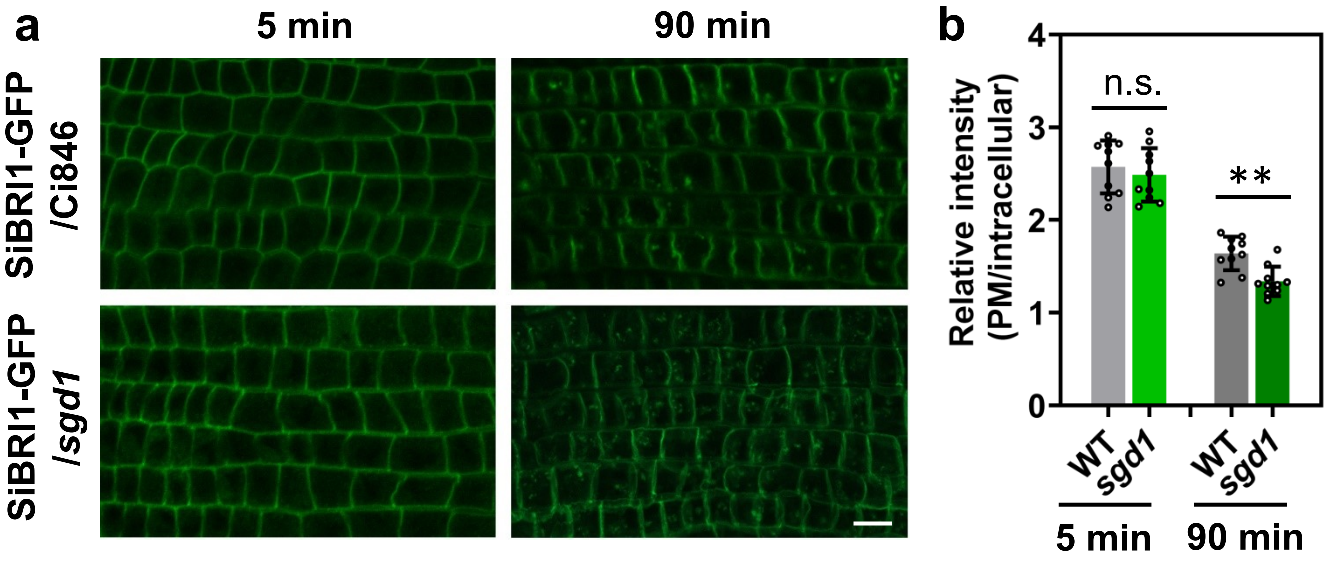

Supplementary Fig. 16 | Analysis of intracellular SiBRI1 in wild-type and *sgd1* plants. a Intracellular SiBRI1 in *sgd1* mutants. SiBRI1-GFP was expressed in wild-type and *sgd1* plants. Five-day-old seedlings were treated with cycloheximide (100 μM) for 5 min and 90 min. Root epidermal cells were observed by confocal microscopy. Bar = 10 μm. **b** Relative fluorescence intensity ratio of plasm membrane (PM) to intracellular related to (**a**) was analyzed using ImageJ (n = 10). Data are means ± SD. $^{**}P < 0.001$ using unpaired two-sided Student's *t*-tests. NS: not statistically significant. Source data are provided as a Source Data file.

Supplementary Tables

Supplementary Table 1. Comparison of major agronomic traits between *sgd1* mutants and the wild-type Yugu1.

Agronomic trait	Yugu1	*sgd1-1*	*sgd1-2*	*n*
Plant height (cm)	130.32±2.81	85.12±1.82**	87.54±2.70**	5
Stem diameter (mm)	7.15±0.02	7.17±0.02	6.21±0.31*	3
Peduncle length (cm)	24.23±2.33	11.00±1.16**	9.77±0.52**	3
Flag leaf length (cm)	40.90±0.86	26.73±0.45**	25.25±0.47**	3
Flag leaf width (cm)	2.50±0.08	2.40±0.07	2.44±0.01	3
Panicle length (cm)	22.02±1.48	15.42±0.70**	14.78±1.32**	5
Panicle diameter (mm)	26.62±0.39	26.08±0.44	23.59±1.54	3
Panicle weight per plant (g)	16.92±1.72	10.63±2.35**	11.03±3.71**	5
Spikelet number	144±7.26	115±3.56**	128±6.34**	3
Grain weight per plant (g)	14.45±1.55	8.60±2.01**	9.12±3.17**	5
1000-grain weight (g)	2.86±0.06	1.65±0.07**	1.63±0.05**	5
Grain length (mm)	1.98±0.10	1.76±0.10**	1.76±0.10**	>200
Grain width (mm)	1.67±0.10	1.32±0.08**	1.35±0.08**	>200
Cell length (μm)	48.75±5.57	41.67±4.81**	43.36±4.59**	>40
Cell width (μm)	34.86±3.32	28.12±3.38**	26.97±2.56**	>40
Cell number in major axis	49.60±1.62	49.60±2.15	49.20±2.04	5
Cell number in minor axis	49.20±1.17	50.40±1.85	49.20±2.48	5

Data are presented as means ±SD *n* indicated biological replicates were measured for each agronomic trait. Asterisks indicate a significant difference between Yugu1 and *sgd1* mutants, unpaired two-sided Student's *t*-tests, *$P < 0.01$, **$P < 0.001$, compared with Yugu1.

Supplementary Table 2. Genetic analysis of F_2 populations from "*sgd1-1* × SSR41" and "*sgd1-2* × Yugu1" crosses.

Combination	F_1 population	F_2 population					
		No. of wild-type phenotype plants	No. of mutant phenotype plants	Ratio	χ2	*P*	α
sgd1-1×SSR41	All wild-type phenotype plants	2571	817	3.15:1	1.42	0.23	0.05
sgd1-2×Yugu1	All wild-type phenotype plants	244	73	3.34:1	0.66	0.42	0.05

Genetic analysis evidenced that both *sgd1-1* and *sgd1-2* were controlled by a single recessive gene.

Supplementary Table 3. The statistics of mutmap sequencing result of *sgd1-1* and *sgd1-2* in the candidate region.

Sample ID	Chromosome position	Reference base	Altered base	Ref/Alt	Type of mutation	Gene ID
sgd1-1	**Chr.9: 7663688**	**A**	**T**	**0/39**	**splice acceptor variant**	**Seita.9G123200**
sgd1-1	Chr.9: 7680049	GAGAG	G	12/7	intergenic region (het)	no gene
sgd1-1	Chr.9: 7685299	TAAA	TAAAA	0/5	intergenic region (hom)	no gene
sgd1-2	**Chr.9: 7665480**	**C**	**T**	**0/38**	**Early stop (R442*)**	**Seita.9G123200**
sgd1-2	Chr.9: 7729260	C	A	0/12	intergenic region (hom)	no gene
sgd1-2	Chr.9: 7731116	C	T	33/10	intergenic region (het)	no gene
sgd1-2	Chr.9: 7731292	G	A	26/8	intergenic region (het)	no gene

Supplementary Table 4. Comparison of agronomic traits between wild-type Ci846, *sgd1*, *Siubc32*, and *sgd1*/*Siubc32* double mutants.

**Agronomic trait**	**WT**	***sgd1***	**Comparison (*sgd1* to WT)**	***Siubc32***	**Comparison (*Siubc32* to WT)**	***sgd1/Siubc32***	**Comparison (*sgd1/Siubc32* to WT)**	***n***
Plant height (cm)	59.54±5.39	44.70±0.99**	-24.92%	40.92±1.80**	-31.27%	29.00±1.47**	-51.29%	5
Flag leaf length (cm)	27.68±2.11	20.50±2.48*	-25.93%	18.98±2.14**	-31.43%	13.08±0.34**	-52.75%	5
Panicle length (cm)	7.17±0.56	4.73±0.45**	-34.00%	4.50±0.40**	-37.24%	2.41±0.30**	-66.37%	5
1000-grain weight (g)	2.75±0.04	1.65±0.03**	-40.00%	1.64±0.03**	-40.50%	1.56±0.03**	-43.23%	5
Grain length (mm)	2.47±0.10	2.19±0.09**	-11.36%	2.13±0.08**	-13.72%	2.03±0.12**	-17.50%	>100
Grain width (mm)	1.70±0.07	1.32±0.08**	-22.29%	1.32±0.08**	-22.40%	1.29±0.08**	-24.08%	>100

Data were presented as means ± SD *n* indicated biological replicates were measured for each agronomic trait. Asterisks indicate a significant difference between Ci846 and 3 mutants, unpaired two-sided Student's *t*-tests, *$P < 0.01$, **$P < 0.001$, compared with Ci846.

Supplementary Table 5. Summary of 13 agronomic traits used in haplotype study.

Trait (unit)	Variety number	Minimum	Maximum	Mean	Median	SD	Skewness	Kurtosis	CV	Diversity Index
**Panicle length (cm)**	960	2.00	43.00	21.13	21.50	6.28	-0.24	0.67	0.30	6.79
**Panicle weight (g)**	960	0.14	33.28	11.28	10.96	6.06	0.27	-0.52	0.54	6.59
**Grain weight per plant (g)**	960	2.26	38.16	14.08	10.84	9.63	0.92	-0.11	0.68	3.19
**Panicle width (mm)**	960	8.50	43.40	22.95	22.56	5.38	0.36	0.43	0.23	6.79
**Primary branch number**	960	18.00	247.00	107.57	105.00	28.45	0.66	1.24	0.26	6.70
**Grain weight per panicle (g)**	960	0.12	13.97	4.86	4.39	3.03	0.53	-0.43	0.62	6.50
**Number of grains per panicle**	960	1.00	274.00	56.02	49.00	39.66	1.23	2.32	0.71	5.89
**Number of florets per panicle**	960	13.00	347.00	95.30	90.00	46.13	1.02	1.99	0.48	6.41
**1000-grain weight (g)**	960	0.64	4.53	2.41	2.50	0.58	-0.74	0.89	0.24	6.78
**Grain length (mm)**	960	1.51	2.61	2.10	2.09	0.16	0.19	0.05	0.08	6.78
**Grain width (mm)**	960	0.99	2.05	1.60	1.61	0.13	-1.95	6.36	0.08	6.78
**Grain area (mm^2)**	960	1.43	4.15	2.70	2.71	0.34	-0.65	2.16	0.13	6.77
**Blast resistance**	960	2.00	9.00	6.69	7.00	2.00	-0.73	-0.44	0.30	6.68

Note: SD, Standard Deviation; CV, Coefficient of Variation; Diversity Index is Shannon's Diversity Index.

Supplementary Table 6. Primers used in this study.

Primer ID	Forward Primer (5'→3')	Reverse Primer (5'→3')	Annotation
**b102_fwd**	CCGTGAAACCCACCACTATT	GCACACACAAACCCGTCA	Map-base cloning
**b246_fwd**	CACGCACGTAGTATTGCTAT	GTTCTGGGCTTCTGGCTG	Map-base cloning
**b201_fwd**	CTTCACTGGCTCCAACTCC	TGTCGGTTCCAGCTCTTGT	Map-base cloning
**CAAS9036F**	CGCCGCTCATCCTCTTCCACAC	GTGCCCATGAACGGATCGCACT	Map-base cloning
**MPGC34**	ACGAGTTGGCTGGATAACC	ACCCTGATTTGTGGCTCTT	Map-base cloning
**p37**	CGGGAAGCAAATGTTCAGAC	GCATGAAGCTCGTCGTCTAC	Map-base cloning
**SNP976**	CTTCCGCTGACGCATAGTTC	ACGCCTTGACATCTACCTTGA	Map-base cloning
**SNP977**	GATCGGTCGTGTCATCACAG	TCCTGCTTGGCTGCTTCA	Map-base cloning
**CAAS9033**	GTGAACCTGCGAATCTCGGGGC	ACGAACGAACGCGAACACACAC	Map-base cloning
**crispr-sisgd1**	TGCTGCGACAATCGAATGACgttttagagctagaaat	GTCATTCGATTGTCGCAGCACggcagccaagccagca	CRISPR/Cas9 genome editing
**crispr-ossgd1**	ATTTTCCTGACTACCTCAGGgttttagagctagaaat	CCTGAGGTAGTCAGGAAAATggcagccaagccagca	CRISPR/Cas9 genome editing
**crispr-tasgd1A/B/D**	ATTGGAATGTAGCGAGCGCTgttttagagctagaaat	AGCGCTCGCTACATTCCAATtgcttcttggtgccgcgcctcc	CRISPR/Cas9 genome editing
**crispr-siubc32**	TTTGAGGGTGGAATCTACCAgttttagagctagaaat	TGGTAGATTCCACCCTCAAAggcagccaagccagca	CRISPR/Cas9 genome editing
**Com-SiSGD1**	aattcgagctcgctaggtaccTGTGTAGCGAGGTGGCTTGAT	gcccttgctcaccatggatccAACATCATATACAGGCATGCGC	pSGD1::SGD1-eGFP
**Com-ZmSGD1**	gttacttctgcactaggtaccATGCAGCGGCGGCGGGGGCA	gcccttgctcaccatggatccAACATCATATACAGGCATGCGT	pUbi::ZmSGD1-eGFP
**OE-SiSGD1H1-eGFP**	TTACTTCTGCACTAGGTACCATGCAGCGGCGGCGG	GGACTTAAGACTAGTAACATCATATACAGGCATGCGC	pUbi-SiSDG1-GFP

**OE-SiBRI1**	GTTACTTCTGCACTAGGTACCATGGAATCTCCGGGGCTG	GCCCTTGCTCACCATGGATCCATCCTTCTCCTCCTTGTCTTCATTC	pUbi-SiBRI1-GFP
**pSiSGD1-GUS**	GCAGGCATGCAAGCTTAATGACTGTGGAGGAATGGACG	CTCAGATCTACCATGG CAAGTGCTCGTGGAAGCCGA	cloned to pCAMBIA1305.1-GUS-plus
**35S-SiSGD1-GFP**	CTTGCATGCCTGCAGGTCGACATGCAGCGGCGGCGGGGG	GCCCTTGCTCACCATGGATCCAACATCATATACAGGCATGCGC	Subcellular localization
**35S-SiUBC32-RFP**	aagtccggagctagctctagaATGGCGGCCACGGCGAAG	ggtcctcgagacgtctctagaGAACTTGCCCTCAATGAAACCT	Subcellular localization
**ENTRY-SiSGD1**	AAAAGCAGGCTCCGAATTCATGCAGCGGCGGCGGGGGCAAA	AGAAAGCTGGGTCGAATTCAACATCATATACAGGCATGCGCT	cloned into pCR™8/GW/TOPO for gwpMAL-C2 vector construction
**ENTRY-SiUBC32**	AAAAGCAGGCTCCGAATTCATGGCGGCCACGGCGAAGTA	AGAAAGCTGGGTCGAATTCGAACTTGCCCTCAATGAAACC	cloned into pCR™8/GW/TOPO for gwpGEX4T-1 vector construction
**HBT-SiSGD1-FLAG**	CCTTGCTCCGTGGATCCATGCAGCGGCGGCGGGGGCAAA	TTGTAGTCAGAAGGCCTAACATCATATACAGGCATGCGCT	Co-IP
**HBT-SiBRI1-HA**	CCTTGCTCCGTGGATCCATGGAATCTCCGGGGCTGC	TCGTATGGGTAAGGCCTATCCTTCTCCTCCTTGTCTTC	Co-IP
**HBT-SiUBC32-FLAG**	CCTTGCTCCGTGGATCCATGGCGGCCACGGCGAAGTA	TTGTAGTCAGAAGGCCTGAACTTGCCCTCAATGAAACC	Co-IP
**HBT-SiSGD1-HA**	CCTTGCTCCGTGGATCCATGCAGCGGCGGCGGGGGCAAA	TCGTATGGGTAAGGCCTAACATCATATACAGGCATGCGCT	Co-IP
**pCDFDuet-MBP-BRI1c-HA-UBA1-S**	GAGGGAAGGATTTCAGAATTCATGGGACTCTTGTTGTCACTGTTCT	CGAAGATTCCTGTGACCTGCAGGATCCTTCTCCTCCTTGTCTTCATTC	Ubiquitination system in the reconstituted bacteria
**pACYCDuet-SiSGD1c-MYC**	CACCAAGCCAGGGATCCATGTCGTCATCAGCAGCAATTAG	AGCTTCTGCTCAGGCCTAACATCATATACAGGCATGCGC	Ubiquitination system in the reconstituted bacteria
**pACYCDuet-SiUBC32-S**	taagaaggagatatacatatgGCGGCCACGGCGAAGTAC	ccaattgagatctgccatatgGAACTTGCCCTCAATGAAACCT	Ubiquitination system in the reconstituted bacteria

**pACYCDuet-SiE2CK-S**	taagaaggagatatacatatgTCGTCACCAAGCAAGCGCCGC	ccaattgagatctgccatatgAGGATCGGGATTGCCGAG	Ubiquitination system in the reconstituted bacteria
**SiSGD1-NLUC**	gagctcggtacccggggatccATGCAGCGGCGGCGGGGGCA	gcgtacgagatctggtcgacAACATCATATACAGGCATGCG	Split-LUC
**CLUC-SiBIN2**	ggggcggtacccggggatccATGGAGGCGCCGCCGGGGCCG	cgaaagctctgcaggtcgacCTAGCTTCCAGCATGCCCAAAG	Split-LUC
**CLUC-SiBRI1**	ggggcggtacccggggatccATGGAATCTCCGGGGCTGCTC	cgaaagctctgcaggtcgacCTAATCCTTCTCCTCCTTGTC	Split-LUC
**CLUC-SiBAS1**	ggggcggtacccggggatccATGGGGTGGTGCTGGGCGGC	cgaaagctctgcaggtcgacTCACATGCCCCGATTATGATCG	Split-LUC
**CLUC-SiUBC32**	ggggcggtacccggggatccATGGCGGCCACGGCGAAGTA	cgaaagctctgcaggtcgacTCAGAACTTGCCCTCAATGAAAC	Split-LUC
**pBT3-SUC-SiSDG1**	attaacaaggccattacggccCAGCGGCGGCGGGGGCAAAC	aactgattggccgaggcggccccAACATCATATACAGGCATGCGC	Y2H
**PPR3N-SiUBC32**	agattacgctggatccATGGCGGCCACGGCGAAGTA	accactgcttggatccTCAGAACTTGCCCTCAATGAAAC	Y2H
**PPR3N-SiBRI1**	agattacgctggatccATGGATTCCTTGTGGGCAG	accactgcttggatccCTAATCCTTCTCCTCCTTGGC	Y2H
**PPR3N-SiBRI1n**	agattacgctggatccATGGATTCCTTGTGGGCAG	accactgcttggatccCTACAGCTGATTATTCGAAAGGTTG	Y2H
**PPR3N-SiBRI1c**	agattacgctggatccATGGGACTCTTGTTGTCACTG	accactgcttggatccCTAATCCTTCTCCTCCTTGGC	Y2H
**qPCR-SiD2**	AGGTGGAAGGAGAAGGACAC	TTGGGAAGTTGACGATGTG	qRT-PCR
**qPCR-SiCYP51G3**	CCGCATGTCGTTAGCCTTGT	TGACGTGCAGTTAGCAAGGAC	qRT-PCR
**qPCR-SiGLR3.7**	TGCAGGACCCATCATCGCTA	TGCAAATTACAGGCGAACCG	qRT-PCR
**qPCR-SiCBF2**	GCCTCAACTTCGCCGACTC	GTCCCAGCTCATGTCGTTGA	qRT-PCR

**qPCR-SiBRH1**	CCTGCTCCTTCTCGGGTACA	GAGCACCTCCTCGATGACCA	qRT-PCR
**qPCR-SiCullin**	ACTCCTCCTCCTCGCCGCCG	CACCTCTGCACTAGCTCCCT	qRT-PCR
**qPCR-SiActin**	GACGCACAACAGGTATCGTG	TCAAGTGCCACATAGGCGAG	qRT-PCR
**35S-SiBRI1-GFP**	cactaggtacctgcaactagtATGGAATCTCCGGGG CTG	agctccggacttaagactagtATCCTTCTCCTCCTTGT CTTCATTC	test the specificity of SiBRI1 antibody
**35S-SiBRI1.L1-GFP**	cactaggtacctgcaactagtATGGACATGCTCCAC GTCTTG	agctccggacttaagactagtGTCGCAAGCAGCGGA CTG	test the specificity of SiBRI1 antibody
**35S-SiBRI1.L2-GFP**	cactaggtacctgcaactagtATGGCCGCCTCCACG ACG	agctccggacttaagactagtTGTTGATTTCTCTGCTG ATTCATCT	test the specificity of SiBRI1 antibody
**35S-SiBRI1.L3-GFP**	cactaggtacctgcaactagtATGGGCGCCGCTCG CATG	agctccggacttaagactagtCACTGATTTCTCAGAT GATTCCTCTATA	test the specificity of SiBRI1 antibody
