# Supplementary material for: An E2-E3 pair contributes to seed size control in grain crops
Source: Nat Commun. 2023 May 29;14:3091. doi: 10.1038/s41467-023-38812-y (PMC10226984; doi:10.1038/s41467-023-38812-y)
Supplement: Supplementary file 3 — Description of Additional Supplementary Files [file 41467_2023_38812_MOESM3_ESM.pdf]

Description of Additional Supplementary Files

Supplementary Data 1. Foxtail millet multi-tissues RNAseq dataset.

Supplementary Data 2. Genome-wide identification of RING type proteins in foxtail millet.

Supplementary Data 3. Blast identity values between SGD1 and other C3HC4 RING-type ubiquitin ligase in different species.

Supplementary Data 4. A list of potential SGD1-interacting proteins identified by the split-ubiquitin membrane yeast two-hybrid (Y2H) screening.

Supplementary Data 5. Gene expression and annotation of potential BZR1-target genes in *sgd1*.

Supplementary Data 6. Gene expression and annotation of co-expressed BZR1-target genes in WT and BR-related mutants.

Supplementary Data 7. Summary of DEGs in the most enriched biological processes of RNAseq.
