# Supplementary material for: An E2-E3 pair contributes to seed size control in grain crops
Source: Nat Commun. 2023 May 29;14:3091. doi: 10.1038/s41467-023-38812-y (PMC10226984; doi:10.1038/s41467-023-38812-y)
Supplement: Supplementary file 5 — Reporting Summary [file 41467_2023_38812_MOESM5_ESM.pdf]

nature portfolio

Corresponding author(s): Xianmin Diao

Last updated by author(s): Apr 16, 2023

Reporting Summary

Nature Portfolio wishes to improve the reproducibility of the work that we publish. This form provides structure for consistency and transparency in reporting. For further information on Nature Portfolio policies, see our Editorial Policies and the Editorial Policy Checklist.

Statistics

For all statistical analyses, confirm that the following items are present in the figure legend, table legend, main text, or Methods section.

n/a	Confirmed	
☐	☒	The exact sample size (*n*) for each experimental group/condition, given as a discrete number and unit of measurement
☐	☒	A statement on whether measurements were taken from distinct samples or whether the same sample was measured repeatedly
☐	☒	The statistical test(s) used AND whether they are one- or two-sided<br>*Only common tests should be described solely by name; describe more complex techniques in the Methods section.*
☒	☐	A description of all covariates tested
☒	☐	A description of any assumptions or corrections, such as tests of normality and adjustment for multiple comparisons
☐	☒	A full description of the statistical parameters including central tendency (e.g. means) or other basic estimates (e.g. regression coefficient) AND variation (e.g. standard deviation) or associated estimates of uncertainty (e.g. confidence intervals)
☐	☒	For null hypothesis testing, the test statistic (e.g. *F*, *t*, *r*) with confidence intervals, effect sizes, degrees of freedom and *P* value noted<br>*Give P values as exact values whenever suitable.*
☒	☐	For Bayesian analysis, information on the choice of priors and Markov chain Monte Carlo settings
☒	☐	For hierarchical and complex designs, identification of the appropriate level for tests and full reporting of outcomes
☒	☐	Estimates of effect sizes (e.g. Cohen's *d*, Pearson's *r*), indicating how they were calculated

Our web collection on statistics for biologists contains articles on many of the points above.

Software and code

Policy information about availability of computer code

Data collection	Plants were photographed by a Canon EOS 500D camera. Spikelets, florets, and grains were captured using Leica M165FC microscope. Outer lemmas cells were observed by Hitachi S3400N scanning electron microscope. Fluorescence microscopic images were collected on Zeiss LSM700 confocal microscope. Luciferase complementation arrays were observed by using Berthold NightSHADE LB 985 plant imaging system. Western blot images were captured by Vilber GmbH FUSION Solo S Imaging system.
Data analysis	Data and statistics were analyzed by GraphPad Prism8 (GraphPad Software) and R (version 4.2.1). Photos and images were analyzed and processed by ZEN Microscopy Software (Zeiss) and ImageJ (Version 1.49). Selective sweep analysis was done by VCFtools (http://vcftools.sourceforge.net/). Haplotype and association analysis were performed by an in-house R script available at https://github.com/ZhangRenL/geneHapR.

For manuscripts utilizing custom algorithms or software that are central to the research but not yet described in published literature, software must be made available to editors and reviewers. We strongly encourage code deposition in a community repository (e.g. GitHub). See the Nature Portfolio guidelines for submitting code & software for further information.

Data

Policy information about availability of data

All manuscripts must include a data availability statement. This statement should provide the following information, where applicable:

- Accession codes, unique identifiers, or web links for publicly available datasets
- A description of any restrictions on data availability
- For clinical datasets or third party data, please ensure that the statement adheres to our policy

Data availability: The high-throughput sequencing data generated in this study were deposited into China National Center for Bioinformation CNCB with accession numbers CRA007999 [https://ngdc.cncb.ac.cn/gsa/browse/CRA007999] and CRA008001 [https://ngdc.cncb.ac.cn/gsa/browse/CRA008001] which are publicly accessible. Gene sequence information of foxtail millet, maize, rice, and Arabidopsis from this study can be found in Phytozome v13 (Setaria italica v2.2, Zea mays RefGen_V4, Oryza sativa v7.0), TAIR, or NCBI, under the following accession numbers: SGD1 (Seita.9G123200) [https://phytozome-next.jgi.doe.gov/report/gene/Sitalica_v2_2/Seita.9G123200], SiUBC32 (Seita.9G428900) [https://phytozome-next.jgi.doe.gov/report/gene/Sitalica_v2_2/Seita.9G428900], SiE2CK (Seita.9G236200) [https://phytozome-next.jgi.doe.gov/report/gene/Sitalica_v2_2/Seita.9G236200], SiBZR1 (Seita.2G367800) [https://phytozome-next.jgi.doe.gov/report/gene/Sitalica_v2_2/Seita.2G367800], SiBAS1 (Seita.5G123900) [https://phytozome-next.jgi.doe.gov/report/gene/Sitalica_v2_2/Seita.5G123900], SiBIN2 (Seita.5G145300) [https://phytozome-next.jgi.doe.gov/report/gene/Sitalica_v2_2/Seita.5G145300], SiCullin (Seita.3G037700) [https://phytozome-next.jgi.doe.gov/report/gene/Sitalica_v2_2/Seita.3G037700], SiD2 (Seita.5G139200) [https://phytozome-next.jgi.doe.gov/report/gene/Sitalica_v2_2/Seita.5G139200], SiCYP51G3 (Seita.2G356300) [https://phytozome-next.jgi.doe.gov/report/gene/Sitalica_v2_2/Seita.2G356300], SiGLR2.7 (Seita.1G009400) [https://phytozome-next.jgi.doe.gov/report/gene/Sitalica_v2_2/Seita.1G009400], SiCBF2 (Seita.2G280200) [https://phytozome-next.jgi.doe.gov/report/gene/Sitalica_v2_2/Seita.2G280200], SiBRH1 (Seita.7G209400) [https://phytozome-next.jgi.doe.gov/report/gene/Sitalica_v2_2/Seita.7G209400], SiBRI1 (NCBI Gene ID: LOC101765569) [https://www.ncbi.nlm.nih.gov/gene/101765569], SiBRI1.L1 (Seita.9G296000) [https://phytozome-next.jgi.doe.gov/report/gene/Sitalica_v2_2/Seita.9G296000], SiBRI1.L2 (Seita.2G165600) [https://phytozome-next.jgi.doe.gov/report/gene/Sitalica_v2_2/Seita.2G165600], and SiBRI1.L3 (Seita.6G117300) [https://phytozome-next.jgi.doe.gov/report/gene/Sitalica_v2_2/Seita.6G117300], ZmSGD1 (Zm00001d013466) [https://phytozome-next.jgi.doe.gov/report/gene/Zmays_RefGen_V4/Zm00001d013466], OsSGD1 (TT3.1, Os03g49900) [https://phytozome-next.jgi.doe.gov/report/gene/Osativa_v7_0/LOC_Os03g49900], and UBA1 (AT2G30110) [https://www.arabidopsis.org/servlets/TairObject?id=34701&type=locus]. Gene sequence information of wheat is available at Ensembl Plants with accession numbers: TaSGD1A (TraesCS4A02G271200) [http://plants.ensembl.org/Triticum_aestivum/Gene/Summary?g=TraesCS4A02G271200], TaSGD1B (TraesCS4B02G042900) [http://plants.ensembl.org/Triticum_aestivum/Gene/Summary?g=TraesCS4B02G042900], and TaSGD1D (TraesCS4D02G040200) [http://plants.ensembl.org/Triticum_aestivum/Gene/Summary?g=TraesCS4D02G040200]. Seed stocks for all plant materials are available by contacting co-authors Sha Tang and Xianmin Diao. Source data are provided as a Source Data file along with this paper.

Human research participants

Policy information about studies involving human research participants and Sex and Gender in Research.

Reporting on sex and gender	N/A
Population characteristics	N/A
Recruitment	N/A
Ethics oversight	N/A

Note that full information on the approval of the study protocol must also be provided in the manuscript.

Field-specific reporting

Please select the one below that is the best fit for your research. If you are not sure, read the appropriate sections before making your selection.

☒ Life sciences ☐ Behavioural & social sciences ☐ Ecological, evolutionary & environmental sciences

For a reference copy of the document with all sections, see nature.com/documents/nr-reporting-summary-flat.pdf

Life sciences study design

All studies must disclose on these points even when the disclosure is negative.

Sample size	Sample sizes were chosen empirically from basial experiment requirements in plant science and previous experimental experience with similar assays, and/or from sizes generally employed in the field. No statistical approach was used to predetermine sample size.
Data exclusions	No data was excluded from analysis in our experiments.
Replication	All attempts to replicate the experiments in this study were successful. Experimental findings have been reproduced independently by at least two co-authors of this manuscript under the same experimental conditions.
Randomization	Plant individuals of different genotypes or positive transgenic lines were collected randomly for all measurements and experiments in this study.

Blinding	Researchers were not blinded to plant genotypes when we grew and collected different plant materials. For replications of phenotyping and molecular biology related experiments, researchers were blinded to test if the research findings can be reproduced. We also arranged at least two researchers to observe/assessed results independently to confirm our experiment results are not subjective.

Reporting for specific materials, systems and methods

We require information from authors about some types of materials, experimental systems and methods used in many studies. Here, indicate whether each material, system or method listed is relevant to your study. If you are not sure if a list item applies to your research, read the appropriate section before selecting a response.

Materials & experimental systems

n/a	Involved in the study
☐	☒ Antibodies
☒	☐ Eukaryotic cell lines
☒	☐ Palaeontology and archaeology
☒	☐ Animals and other organisms
☒	☐ Clinical data
☒	☐ Dual use research of concern

Methods

n/a	Involved in the study
☒	☐ ChIP-seq
☒	☐ Flow cytometry
☒	☐ MRI-based neuroimaging

Antibodies

Antibodies used	The following antibodies were used for western blot, immunoprecipitation:<br>Anti-FLAG (# F1804) was purchased from Sigma-Aldrich (St Louis, USA).<br>Anti-MBP (#HT701) and anti-GST (#HT601) were purchased from TransGen Biotech (Beijing, China).<br>anti-GFP (#M20004), anti-Myc (#M20002) and anti-HA (#M20003) were purchased from Abmart (Shanghai, China).<br>Anti-S (#ab183674) was purchased from Abcam (Cambridge, UK).<br>Anti-Actin (#AC009) was purchased from ABclonal (Beijing, China).<br>Anti-SiBRI1 and anti-SiBZR1 were prepared by Beijing Protein Innovation Co., Ltd (Beijing, China).<br>Protein-G-agarose beads (#20397), Glutathione SepharoseTM 4B beads (#17075601) and Amylose agarose beads (#E8035) were purchased from Thermo Scientific (Waltham, MA, USA), GE Healthcare (Chicago, IL, USA) and New England Biolabs (Hitchin, Hertfordshire, UK), respectively. The antibodies were applied into western blot at the dilution of 1:3000.
Validation	Validation statements, relevant citations of commercial primary antibodies are available from manufacturers:<br>Anti-FLAG antibody is a mouse monoclonal antibody to the synthetic DYKDDDDK peptide. https://www.sigmaaldrich.cn/CN/zh/product/sigma/f1804.<br>Anti-MBP antibody is a mouse monoclonal antibody to MBP tag. https://www.transgenbiotech.com/tag_antibody/proteinfind_anti_mbp_mouse_monoclonal_antibody.html.<br>Anti-GST antibody is a mouse monoclonal antibody to Glutathione S-Transferase (GST). https://www.transgenbiotech.com/tag_antibody/proteinfind_anti_gst_mouse_monoclonal_antibody.html.<br>Anti-GFP antibody is a mouse monoclonal antibody to full length recombinant GFP. http://www.ab-mart.com.cn/page.aspx?node=60&id=971.<br>Anti-MYC antibody is a mouse monoclonal antibody to a synthetic peptide (KLH-coupled) corresponding to residues 410-419 of human c-Myc (EQKLISEEDL). http://www.ab-mart.com.cn/page.aspx?node=60&id=962.<br>Anti-HA antibody is a mouse monoclonal antibody to the synthetic YPYDVPDYA (KLH-coupled). http://www.ab-mart.com.cn/page.aspx?node=60&id=963.<br>Anti-S antibody is a rabbit polyclonal antibody to the synthetic KETAAAKFERQHMDS peptide. https://www.abcam.com/S-tag-antibody-ab183674.html.<br>Anti-Actin antibody is a mouse monoclonal antibody to recombinant protein of Actin (plant specific). https://abclonal.com.cn/catalog/AC009.<br>Anti-SiBRI1 antibody are rabbit polyclonal antibody from purified proteins which validated and used in our previous publication (doi: 10.1073/pnas.2002278117).<br>Anti-SiBZR1 antibody are rabbit polyclonal antibody from purified proteins which validated and used in our previous publication (doi: 10.1080/15592324.2021.1976561).<br>Protein-G-agarose is an affinity resin for purification antibodies and immunoprecipitation. Samples containing IgG are incubated with Protein G agarose in a buffer that facilitates binding. https://www.thermofisher.cn/order/catalog/product/20397?SID=srch-srp-20397.<br>Glutathione Sepharose 4B is designed for high capacity single-step purification of Glutathione S-Transferase (GST) tagged fusion proteins. https://www.fishersci.com/shop/products/ge-healthcare-glutathione-sepharose-4b-media-3/45000139.<br>Amylose agarose beads is designed for high capacity single-step purification of MBP-fusion proteins. https://www.neb.com/products/e8035-amylose-magnetic-beads#Product%20Information.
